# Supplementary material for: Geographical variations in maternal dietary patterns during pregnancy associated with birth weight in Shaanxi province, Northwestern China
Source: PLoS One. 2021 Jul 22;16(7):e0254891. doi: 10.1371/journal.pone.0254891 (PMC8297908; doi:10.1371/journal.pone.0254891)
Supplement: S1 Table — (DOCX) [file pone.0254891.s001.docx]

Table 1 The association of diet pattern with abnormal birth outcomes using non-spatial logistical regression in South Shaanxi^*^

| Study variable | LBW | Macrosomia | SGA | LGA |
| --- | --- | --- | --- | --- |
| *Sociodemographic characteristics†* |  |  |  |  |
| Child gender(ref= Female) |  |  |  |  |
| Male(1=yes,0=no) | 0.870(0.570-1.328) | 1.553(1.091-2.212) | 1.032(0.789-1.351) | 1.097(0.833-1.445) |
| Fetal number(ref=Singleton) |  |  |  |  |
| Twin and multi-fetal(1=yes,0=no) | 27.617(10.830-70.430) | 0.681(0.145-3.203) | 8.816(3.776-20.585) | 1.646(0.611-4.438) |
| Infant parity(ref=one) |  |  |  |  |
| 2(1=yes,0=no) | 0.945(0.527-1.696) | 1.676(1.063-2.643) | 0.761(0.521-1.112) | 1.315(0.912-1.896) |
| ≥3(1=yes,0=no) | 1.235(0.342-4.454) | 3.101(1.147-8.383) | 0.718(0.269-1.915) | 2.112(0.899-4.960) |
| Childbearing age(ref=18-24) |  |  |  |  |
| 25-29(1=yes,0=no) | 0.807(0.480-1.355) | 1.002(0.640-1.567) | 0.671(0.481-0.937) | 1.110(0.777-1.586) |
| ≥30(1=yes,0=no) | 0.801(0.406-1.582) | 1.045(0.609-1.791) | 0.730(0.470-1.133) | 1.224(0.791-1.892) |
| Mother’s education(ref= Primary school and below) |  |  |  |  |
| Junior high school(1=yes,0=no) | 2.637(1.008-6.898) | 1.455(0.799-2.648) | 0.724(0.476-1.102) | 1.422(0.891-2.272) |
| Senior high school(1=yes,0=no) | 2.506(0.877-7.160) | 1.615(0.796-3.277) | 0.643(0.388-1.065) | 1.119(0.633-1.978) |
| College and above(1=yes,0=no) | 2.782(0.798-9.697) | 1.369(0.527-3.555) | 0.627(0.311-1.265) | 1.082(0.510-2.294) |
| Mother's residence during pregnancy(ref=Permanent) |  |  |  |  |
| Floating(1=yes,0=no) | 1.005(0.546-1.850) | 1.141(0.701-1.858) | 0.905(0.599-1.367) | 1.114(0.750-1.655) |
| Household wealth Index (ref= Poor) |  |  |  |  |
| Middle-income(1=yes,0=no) | 0.735(0.417-1.294) | 1.129(0.714-1.787) | 0.938(0.661-1.332) | 1.155(0.795-1.678) |
| Rich(1=yes,0=no) | 0.938(0.558-1.574) | 1.121(0.720-1.746) | 0.956(0.682-1.340) | 1.231(0.862-1.758) |
| Altitude(ref=less than 500) |  |  |  |  |
| 500-1000(1=yes,0=no) | 1.110(0.682-1.807) | 1.133(0.762-1.684) | 1.380(1.004-1.896) | 0.845(0.626-1.140) |
| >1000(1=yes,0=no) | —— | —— | —— | —— |
| *Dietary patterns during pregnancy* |  |  |  |  |
| Equilibrium pattern(ref=T2) |  |  |  |  |
| T1(1=yes,0=no) | 1.325(0.766-2.290) | 1.037(0.675-1.595) | 0.768(0.550-1.073) | 0.937(0.664-1.322) |
| T3(1=yes,0=no) | 1.112(0.638-1.936) | 0.870(0.560-1.350) | 0.775(0.553-1.086) | 0.837(0.591-1.186) |
| Snacks pattern(ref= T2) |  |  |  |  |
| T1(1=yes,0=no) | 0.869(0.481-1.572) | 2.028(1.223-3.364) | 0.958(0.659-1.393) | 1.613(1.096-2.374) |
| T3(1=yes,0=no) | 1.101(0.665-1.823) | 2.148(1.371-3.365) | 1.202(0.871-1.660) | 1.568(1.108-2.219) |
| Prudent pattern(ref= T2) |  |  |  |  |
| T1(1=yes,0=no) | 1.272(0.702-2.303) | 1.020(0.654-1.592) | 0.931(0.642-1.350) | 0.886(0.612-1.282) |
| T3(1=yes,0=no) | 1.376(0.820-2.310) | 0.760(0.501-1.152) | 1.152(0.835-1.588) | 0.844(0.608-1.171) |

T, tertiles; LBW, low birth weight; SGA, small for gestational age; LGA, large for gestational age.

^*^ Values are OR of abnormal birth outcomes and its 95% confidence interval is included in the bracket.

^†^ OR are adjusted for socio-demographic characteristics (child gender, fetal number, infant parity, childbearing age, mother’s education, mother's residence during pregnancy, Household wealth Index, altitude of residence and area).
