# Supplementary material for: Geographical variations in maternal dietary patterns during pregnancy associated with birth weight in Shaanxi province, Northwestern China
Source: PLoS One. 2021 Jul 22;16(7):e0254891. doi: 10.1371/journal.pone.0254891 (PMC8297908; doi:10.1371/journal.pone.0254891)
Supplement: S2 Table — (DOCX) [file pone.0254891.s002.docx]

Table 2 The association of diet pattern with abnormal birth outcomes using non-spatial logistical regression in Central Shaanxi^*^

| Study variable | LBW | Macrosomia | SGA | LGA |
| --- | --- | --- | --- | --- |
| *Sociodemographic characteristics†* |  |  |  |  |
| Child gender(ref= Female) |  |  |  |  |
| Male(1=yes,0=no) | 0.683(0.481-0.970) | 1.641(1.250-2.155) | 0.941(0.771-1.149) | 1.007(0.816-1.243) |
| Fetal number(ref=Singleton) |  |  |  |  |
| Twin and multi-fetal(1=yes,0=no) | 39.868(18.319-86.764) | —— | 7.758(3.966-15.175) | 0.411(0.096-1.749) |
| Infant parity(ref=one) |  |  |  |  |
| 2(1=yes,0=no) | 0.802(0.518-1.243) | 1.254(0.894-1.758) | 0.874(0.678-1.127) | 1.387(1.062-1.811) |
| ≥3(1=yes,0=no) | 0.768(0.305-1.934) | 1.131(0.488-2.619) | 0.932(0.531-1.635) | 1.181(0.596-2.340) |
| Childbearing age(ref=18-24) |  |  |  |  |
| 25-29(1=yes,0=no) | 1.808(1.127-2.900) | 1.034(0.719-1.488) | 1.108(0.857-1.433) | 1.062(0.797-1.416) |
| ≥30(1=yes,0=no) | 2.013(1.130-3.587) | 1.542(1.017-2.340) | 1.155(0.839-1.589) | 1.429(1.024-1.994) |
| Mother’s education(ref= Primary school and below) |  |  |  |  |
| Junior high school(1=yes,0=no) | 0.520(0.275-0.984) | 0.684(0.369-1.268) | 0.799(0.529-1.209) | 1.204(0.676-2.144) |
| Senior high school(1=yes,0=no) | 0.679(0.336-1.371) | 0.877(0.456-1.690) | 0.819(0.520-1.291) | 1.348(0.735-2.472) |
| College and above(1=yes,0=no) | 0.290(0.121-0.692) | 1.145(0.574-2.284) | 0.598(0.357-1.000) | 1.712(0.909-3.225) |
| Mother's residence during pregnancy(ref=Permanent) |  |  |  |  |
| Floating(1=yes,0=no) | 1.044(0.646-1.687) | 1.214(0.847-1.739) | 0.917(0.692-1.216) | 1.237(0.933-1.642) |
| Household wealth Index (ref= Poor) |  |  |  |  |
| Middle-income(1=yes,0=no) | 1.110(0.724-1.700) | 1.268(0.904-1.779) | 0.947(0.745-1.204) | 1.159(0.888-1.512) |
| Rich(1=yes,0=no) | 1.148(0.727-1.811) | 1.216(0.855-1.731) | 0.893(0.688-1.159) | 1.220(0.926-1.607) |
| Altitude(ref=less than 500) |  |  |  |  |
| 500-1000(1=yes,0=no) | 0.987(0.639-1.527) | 0.782(0.576-1.062) | 1.153(0.896-1.485) | 1.135(0.892-1.445) |
| >1000(1=yes,0=no) | 1.721(1.070-2.769) | 0.637(0.424-0.957) | 2.230(1.699-2.926) | 0.788(0.569-1.093) |
| *Dietary patterns during pregnancy* |  |  |  |  |
| Equilibrium pattern(ref=T2) |  |  |  |  |
| T1(1=yes,0=no) | 1.039(0.680-1.588) | 1.112(0.781-1.584) | 1.052(0.824-1.344) | 1.029(0.777-1.363) |
| T3(1=yes,0=no) | 0.784(0.492-1.248) | 1.334(0.962-1.850) | 0.683(0.527-0.885) | 1.316(1.015-1.706) |
| Snacks pattern(ref= T2) |  |  |  |  |
| T1(1=yes,0=no) | 0.707(0.439-1.140) | 0.925(0.660-1.297) | 1.097(0.844-1.427) | 1.010(0.773-1.320) |
| T3(1=yes,0=no) | 1.567(1.036-2.370) | 1.027(0.722-1.460) | 1.483(1.153-1.907) | 0.942(0.707-1.253) |
| Prudent pattern(ref= T2) |  |  |  |  |
| T1(1=yes,0=no) | 0.502(0.319-0.792) | 0.825(0.586-1.163) | 1.045(0.814-1.343) | 0.844(0.642-1.111) |
| T3(1=yes,0=no) | 0.556(0.358-0.862) | 0.845(0.614-1.162) | 0.878(0.682-1.131) | 0.910(0.705-1.174) |

T, tertiles; LBW, low birth weight; SGA, small for gestational age; LGA, large for gestational age.

^*^ Values are OR of abnormal birth outcomes and its 95% confidence interval is included in the bracket.

^†^ OR are adjusted for socio-demographic characteristics (child gender, fetal number, infant parity, childbearing age, mother’s education, mother's residence during pregnancy, Household wealth Index, altitude of residence and area).
