# Supplementary material for: Geographical variations in maternal dietary patterns during pregnancy associated with birth weight in Shaanxi province, Northwestern China
Source: PLoS One. 2021 Jul 22;16(7):e0254891. doi: 10.1371/journal.pone.0254891 (PMC8297908; doi:10.1371/journal.pone.0254891)
Supplement: S3 Table — (DOCX) [file pone.0254891.s003.docx]

Table 3 The association of diet pattern with abnormal birth outcomes using non-spatial logistical regression in North Shaanxi*

| Study variable | LBW | Macrosomia | SGA | LGA |
| --- | --- | --- | --- | --- |
| *Sociodemographic characteristics†* |  |  |  |  |
| Child gender(ref= Female) |  |  |  |  |
| Male(1=yes,0=no) | 0.781(0.426-1.434) | 1.799(1.139-2.840) | 0.938(0.655-1.342) | 1.234(0.861-1.767) |
| Fetal number(ref=Singleton) |  |  |  |  |
| Twin and multi-fetal(1=yes,0=no) | 28.274(8.963-89.186) | —— | 5.101(1.852-14.055) | —— |
| Infant parity(ref=one) |  |  |  |  |
| 2(1=yes,0=no) | 0.659(0.320-1.360) | 1.891(1.133-3.158) | 0.730(0.482-1.105) | 1.410(0.929-2.142) |
| ≥3(1=yes,0=no) | 0.506(0.120-2.139) | 1.437(0.503-4.108) | 0.610(0.249-1.498) | 1.358(0.582-3.168) |
| Childbearing age(ref=18-24) |  |  |  |  |
| 25-29(1=yes,0=no) | 1.216(0.588-2.516) | 1.279(0.761-2.152) | 0.830(0.545-1.262) | 1.210(0.791-1.850) |
| ≥30(1=yes,0=no) | 1.743(0.720-4.217) | 1.506(0.789-2.877) | 0.888(0.508-1.553) | 1.621(0.952-2.760) |
| Mother’s education(ref= Primary school and below) |  |  |  |  |
| Junior high school(1=yes,0=no) | 1.584(0.654-3.835) | 0.798(0.446-1.429) | 1.383(0.825-2.319) | 0.798(0.486-1.310) |
| Senior high school(1=yes,0=no) | 1.077(0.310-3.739) | 1.027(0.478-2.208) | 0.884(0.442-1.768) | 1.047(0.557-1.968) |
| College and above(1=yes,0=no) | 0.544(0.124-2.394) | 1.381(0.613-3.110) | 0.676(0.306-1.496) | 1.467(0.760-2.829) |
| Mother's residence during pregnancy(ref=Permanent) |  |  |  |  |
| Floating(1=yes,0=no) | 1.508(0.474-4.798) | 0.609(0.184-2.012) | 0.340(0.104-1.114) | 0.727(0.304-1.739) |
| Household wealth Index (ref= Poor) |  |  |  |  |
| Middle-income(1=yes,0=no) | 0.759(0.357-1.614) | 1.135(0.656-1.964) | 0.620(0.401-0.961) | 1.209(0.768-1.903) |
| Rich(1=yes,0=no) | 1.128(0.541-2.353) | 1.689(1.002-2.847) | 0.695(0.448-1.077) | 1.909(1.241-2.935) |
| Altitude(ref=less than 500) |  |  |  |  |
| 500-1000(1=yes,0=no) | 0.912(0.463-1.799) | 0.688(0.427-1.110) | 1.117(0.734-1.699) | 0.853(0.570-1.277) |
| >1000(1=yes,0=no) | —— | —— | —— | —— |
| *Dietary patterns during pregnancy* |  |  |  |  |
| Equilibrium pattern(ref=T2) |  |  |  |  |
| T1(1=yes,0=no) | 0.926(0.469-1.829) | 1.256(0.744-2.121) | 1.188(0.775-1.821) | 0.770(0.501-1.185) |
| T3(1=yes,0=no) | 0.418(0.158-1.102) | 1.191(0.682-2.080) | 1.316(0.825-2.099) | 0.862(0.551-1.349) |
| Snacks pattern(ref= T2) |  |  |  |  |
| T1(1=yes,0=no) | 1.212(0.486-3.025) | 1.100(0.590-2.050) | 0.886(0.510-1.538) | 0.877(0.526-1.463) |
| T3(1=yes,0=no) | 1.051(0.521-2.121) | 1.032(0.626-1.702) | 0.973(0.647-1.463) | 0.950(0.632-1.427) |
| Prudent pattern(ref= T2) |  |  |  |  |
| T1(1=yes,0=no) | 0.895(0.449-1.785) | 0.784(0.480-1.280) | 1.032(0.676-1.578) | 0.802(0.531-1.211) |
| T3(1=yes,0=no) | 0.692(0.233-2.051) | 0.707(0.344-1.453) | 1.104(0.609-2.000) | 0.928(0.530-1.624) |

T, tertiles; LBW, low birth weight; SGA, small for gestational age; LGA, large for gestational age.

^*^ Values are OR of abnormal birth outcomes and its 95% confidence interval is included in the bracket.

^†^ OR are adjusted for socio-demographic characteristics (child gender, fetal number, infant parity, childbearing age, mother’s education, mother's residence during pregnancy, Household wealth Index, altitude of residence and area).
