# Supplementary material for: Geographical variations in maternal dietary patterns during pregnancy associated with birth weight in Shaanxi province, Northwestern China
Source: PLoS One. 2021 Jul 22;16(7):e0254891. doi: 10.1371/journal.pone.0254891 (PMC8297908; doi:10.1371/journal.pone.0254891)
Supplement: S4 Table — (DOCX) [file pone.0254891.s004.docx]

Table 4 The characteristics of dietary patterns among pregnant women (n 7934) in Shaanxi province, Northwest China, 2013^*^

| Study variable | Equilibrium pattern | | Snacks pattern | | Prudent pattern | |
| --- | --- | --- | --- | --- | --- | --- |
|  | β | p | β | p | β | p |
| Child gender(ref= Female) |  |  |  |  |  |  |
| Male(1=yes,0=no) | 0.019 | 0.406 | -0.041 | 0.064 | 0.002 | 0.912 |
| Fetal number(ref=Singleton) |  |  |  |  |  |  |
| Twin and multi-fetal(1=yes,0=no) | 0.007 | 0.948 | -0.191 | 0.067 | 0.215 | 0.034 |
| Infant parity(ref=one) |  |  |  |  |  |  |
| 2(1=yes,0=no) | -0.093 | 0.001 | 0.106 | <0.001 | -0.109 | <0.001 |
| ≥3(1=yes,0=no) | -0.055 | 0.423 | 0.124 | 0.070 | -0.145 | 0.029 |
| Childbearing age(ref=18-24) |  |  |  |  |  |  |
| 25-29(1=yes,0=no) | 0.045 | 0.109 | -0.138 | <0.001 | -0.031 | 0.252 |
| ≥30(1=yes,0=no) | 0.080 | 0.023 | -0.205 | <0.001 | 0.039 | 0.246 |
| Mother’s education(ref=Primary school and below) |  |  |  |  |  |  |
| Junior high school(1=yes,0=no) | 0.102 | 0.014 | -0.005 | 0.896 | 0.057 | 0.151 |
| Senior high school(1=yes,0=no) | 0.243 | <0.001 | -0.121 | 0.009 | 0.160 | <0.001 |
| College and above(1=yes,0=no) | 0.482 | <0.001 | -0.294 | <0.001 | 0.338 | <0.001 |
| Mother's residence during pregnancy(ref=Permanent) |  |  |  |  |  |  |
| Floating(1=yes,0=no) | 0.045 | 0.182 | -0.025 | 0.453 | 0.095 | 0.004 |
| Household wealth Index (ref= Poor) |  |  |  |  |  |  |
| Middle-income(1=yes,0=no) | 0.066 | 0.019 | 0.007 | 0.803 | 0.046 | 0.088 |
| Rich(1=yes,0=no) | 0.108 | <0.001 | 0.025 | 0.375 | 0.106 | <0.001 |
| Altitude(ref=less than 500) |  |  |  |  |  |  |
| 500-1000(1=yes,0=no) | -0.084 | 0.003 | 0.031 | 0.259 | -0.107 | <0.001 |
| >1000(1=yes,0=no) | -0.147 | <0.001 | 0.069 | 0.066 | -0.292 | <0.001 |
| Area(ref= South area) |  |  |  |  |  |  |
| Central area(1=yes,0=no) | -0.095 | 0.001 | -0.159 | <0.001 | -0.177 | <0.001 |
| North area(1=yes,0=no) | -0.113 | 0.006 | 0.125 | 0.002 | -0.550 | <0.001 |

^*^ Values were derived from the multiple linear regression analysis for dietary pattern scores.
