# Supplementary material for: Geographical variations in maternal dietary patterns during pregnancy associated with birth weight in Shaanxi province, Northwestern China
Source: PLoS One. 2021 Jul 22;16(7):e0254891. doi: 10.1371/journal.pone.0254891 (PMC8297908; doi:10.1371/journal.pone.0254891)
Supplement: S5 Table — (DOCX) [file pone.0254891.s005.docx]

**Table5** The association of diet pattern with abnormal birth outcomes using non-spatial logistical regression*

| Study variable | LBW | Macrosomia | SGA | LGA |
| --- | --- | --- | --- | --- |
|  | n (%) | n (%) | n (%) | n (%) |
| Child gender(ref=Female) |  |  |  |  |
| Male(1=yes,0=no) | 0.754  ( 0.591-0.962) | 1.649  (1.358-2.003) | 0.973  (0.841-1.125) | 1.070  (0.920-1.244) |
| Fetal number(ref=Singleton) |  |  |  |  |
| Twin and multi-fetal(1=yes,0=no) | 31.306  ( 18.641-52.576) | 0.263  (0.063-1.091) | 7.401  (4.684-11.695) | 0.687  (0.324-1.457) |
| Infant parity(ref=one) |  |  |  |  |
| 2(1=yes,0=no) | 0.811  (0.594-1.107) | 1.452  (1.146-1.838) | 0.822  (0.682-0.990) | 1.347  (1.115-1.629) |
| ≥3(1=yes,0=no) | 0.778  (0.403-1.500) | 1.526  (0.890-2.617) | 0.820  (0.537-1.252) | 1.393  (0.893-2.174) |
| Childbearing age(ref=18-24) |  |  |  |  |
| 25-29(1=yes,0=no) | 1.267  (0.934-1.717) | 1.087  (0.850-1.391) | 0.891  (0.744-1.066) | 1.114  (0.915-1.356) |
| ≥30(1=yes,0=no) | 1.372  (0.934-2.013) | 1.395  (1.044-1.863) | 0.937  (0.746-1.178) | 1.428  (1.131-1.804) |
| Mother’s education (ref=Primary school and below) |  |  |  |  |
| Junior high school(1=yes,0=no) | 1.177  (0.751-1.843) | 0.942  (0.671-1.323) | 0.911  (0.707-1.174) | 1.133  (0.852-1.506) |
| Senior high school(1=yes,0=no) | 1.253  (0.755-2.079) | 1.147  (0.780-1.686) | 0.836  (0.624-1.121) | 1.175  (0.850-1.623) |
| College and above(1=yes,0=no) | 0.653  (0.348-1.226) | 1.478  (0.968-2.258) | 0.636  (0.447-0.903) | 1.496  (1.052-2.128) |
| Mother's residence during pregnancy (ref=Permanent) |  |  |  |  |
| Floating(1=yes,0=no) | 1.096  (0.770-1.561) | 1.123  (0.851-1.482) | 0.863  (0.689-1.081) | 1.156  (0.928-1.440) |
| Household wealth Index(ref= Poor) |  |  |  |  |
| Middle-income(1=yes,0=no) | 0.924  (0.680-1.255) | 1.223  (0.960-1.558) | 0.870  (0.728-1.040) | 1.174  (0.967-1.426) |
| Rich(1=yes,0=no) | 1.055  (0.777-1.431) | 1.292  (1.014-1.646) | 0.858  (0.714-1.030) | 1.329  (1.096-1.611) |
| Altitude(ref=less than 500) |  |  |  |  |
| 500-1000(1=yes,0=no) | 1.122  (0.820-1.534) | 0.904  (0.719-1.136) | 1.277  (1.054-1.546) | 1.006  (0.836-1.209) |
| >1000(1=yes,0=no) | 1.581  (1.057-2.367) | 0.661  (0.476-0.917) | 2.133  (1.685-2.701) | 0.788  (0.608-1.023) |
| Area(ref= South area) |  |  |  |  |
| Central area(1=yes,0=no) | 0.892  (0.658-1.210) | 0.995  (0.786-1.260) | 1.068  (0.886-1.288) | 1.005  (0.832-1.214) |
| North area(1=yes,0=no) | 0.609  (0.384-0.967) | 1.420  (1.003-2.011) | 0.536  (0.406-0.709) | 1.298  (0.981-1.717) |
| Equilibrium pattern(ref=T2) |  |  |  |  |
| T1(1=yes,0=no) | 1.075  (0.800-1.444) | 1.137  (0.894-1.446) | 0.968  (0.811-1.157) | 0.948  (0.782-1.149) |
| T3(1=yes,0=no) | 0.844  (0.612-1.164) | 1.169  (0.925-1.479) | 0.801  (0.665-0.965) | 1.069  (0.888-1.287) |
| Snacks pattern(ref=T2) |  |  |  |  |
| T1(1=yes,0=no) | 0.783  (0.557-1.100) | 1.183  (0.920-1.523) | 0.996  (0.817-1.215) | 1.124  (0.921-1.373) |
| T3(1=yes,0=no) | 1.226  (0.919-1.635) | 1.265  (1.000-1.602) | 1.260  (1.056-1.505) | 1.102  (0.911-1.334) |
| Prudent pattern(ref=T2) |  |  |  |  |
| T1(1=yes,0=no) | 0.732  (0.537-0.997) | 0.853  (0.674-1.080) | 0.997  (0.829-1.198) | 0.836  (0.690-1.013) |
| T3(1=yes,0=no) | 0.788  (0.581-1.069) | 0.788  (0.623-0.997) | 0.996  (0.828-1.199) | 0.880  (0.730-1.062) |

T, tertiles; LBW, low birth weight; SGA, small for gestational age; LGA, large for gestational age.

* Values are OR of abnormal birth outcomes and its 95% confidence interval is included in the bracket.
