# Supplementary figures and images for: Geographical variations in maternal dietary patterns during pregnancy associated with birth weight in Shaanxi province, Northwestern China
Source: PLoS One. 2021 Jul 22;16(7):e0254891. doi: 10.1371/journal.pone.0254891 (PMC8297908; doi:10.1371/journal.pone.0254891)

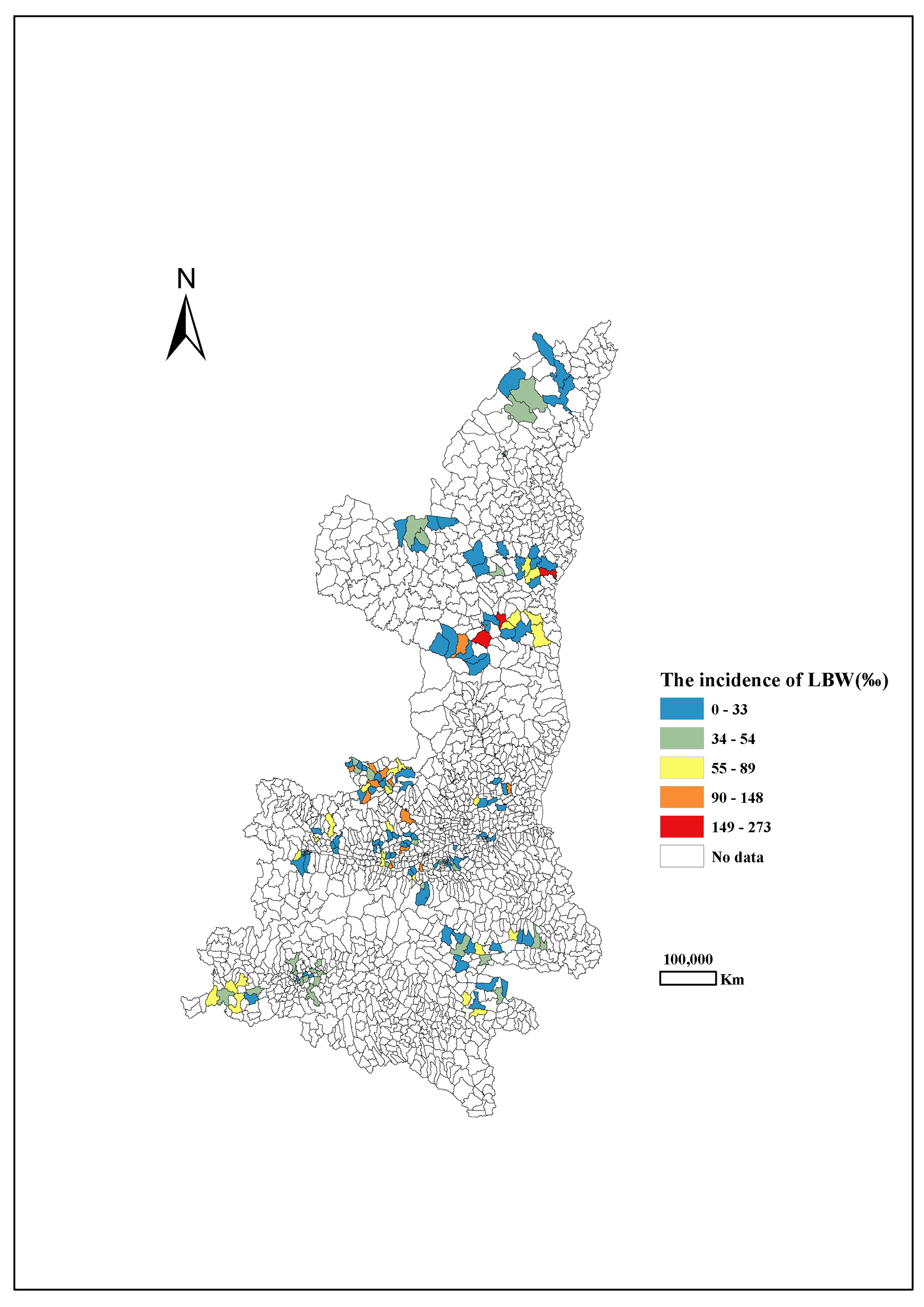

Supplement: S1 Fig — (TIF) [file pone.0254891.s006.tif]

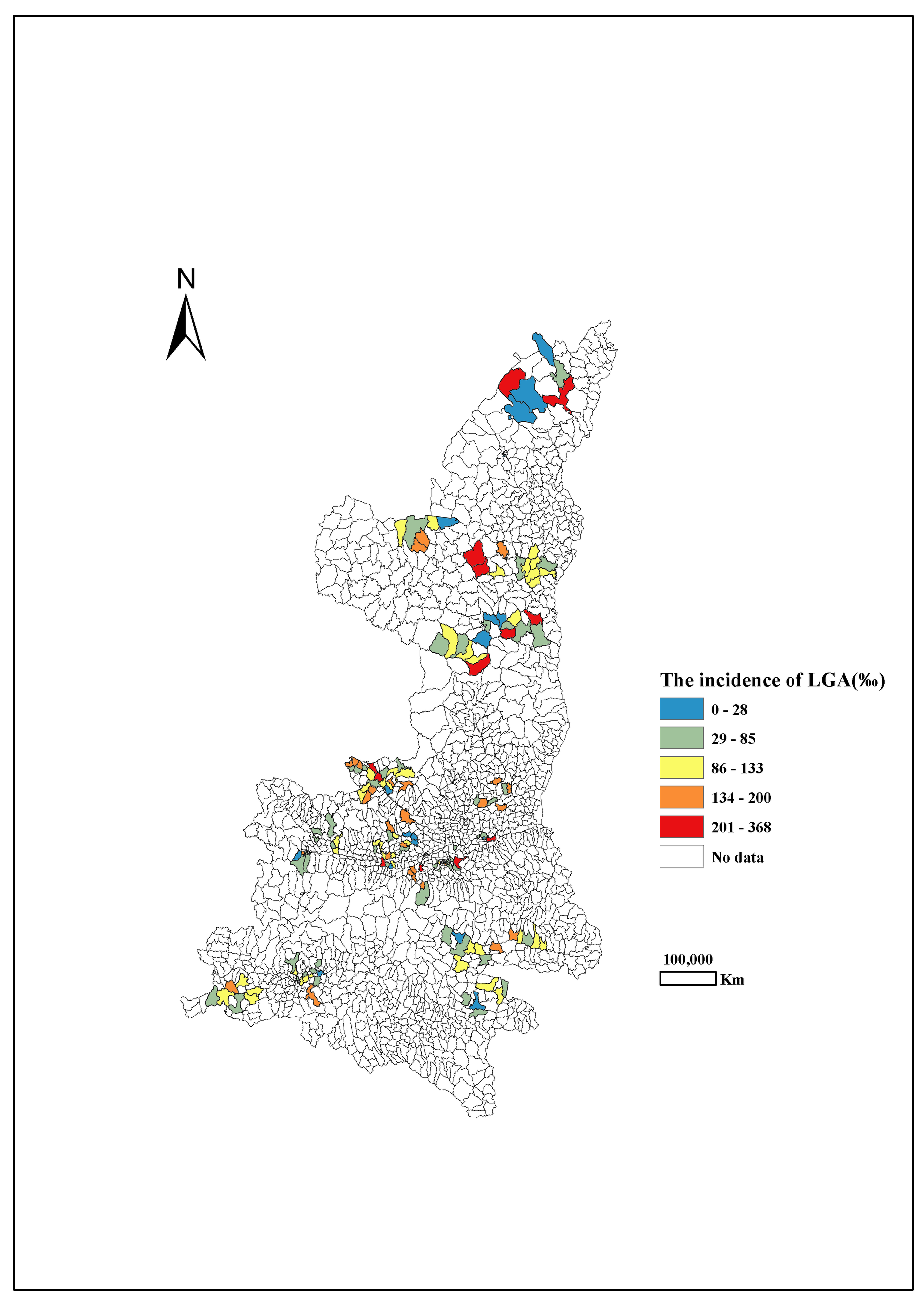

Supplement: S2 Fig — (TIF) [file pone.0254891.s007.tif]

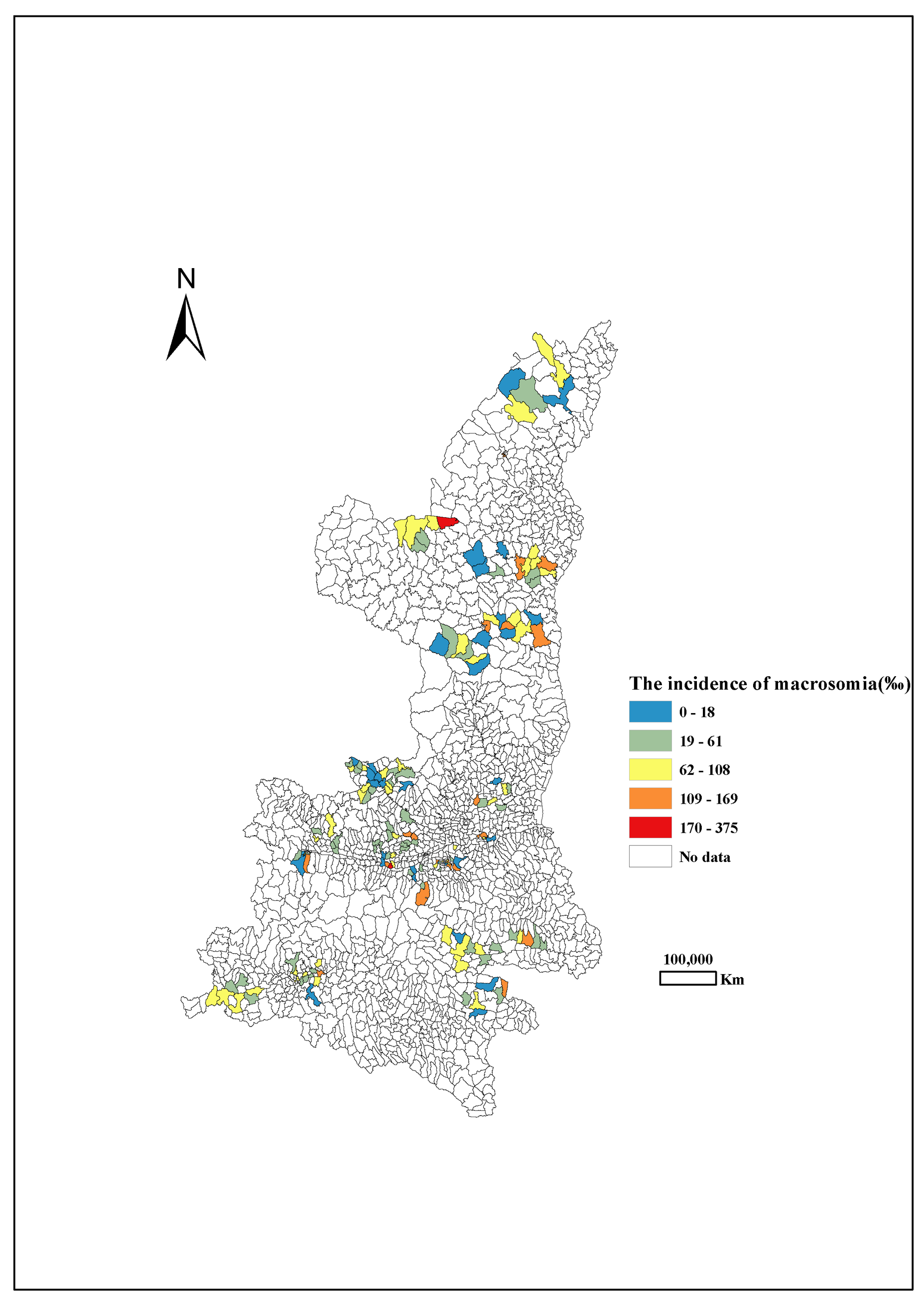

Supplement: S3 Fig — (TIF) [file pone.0254891.s008.tif]

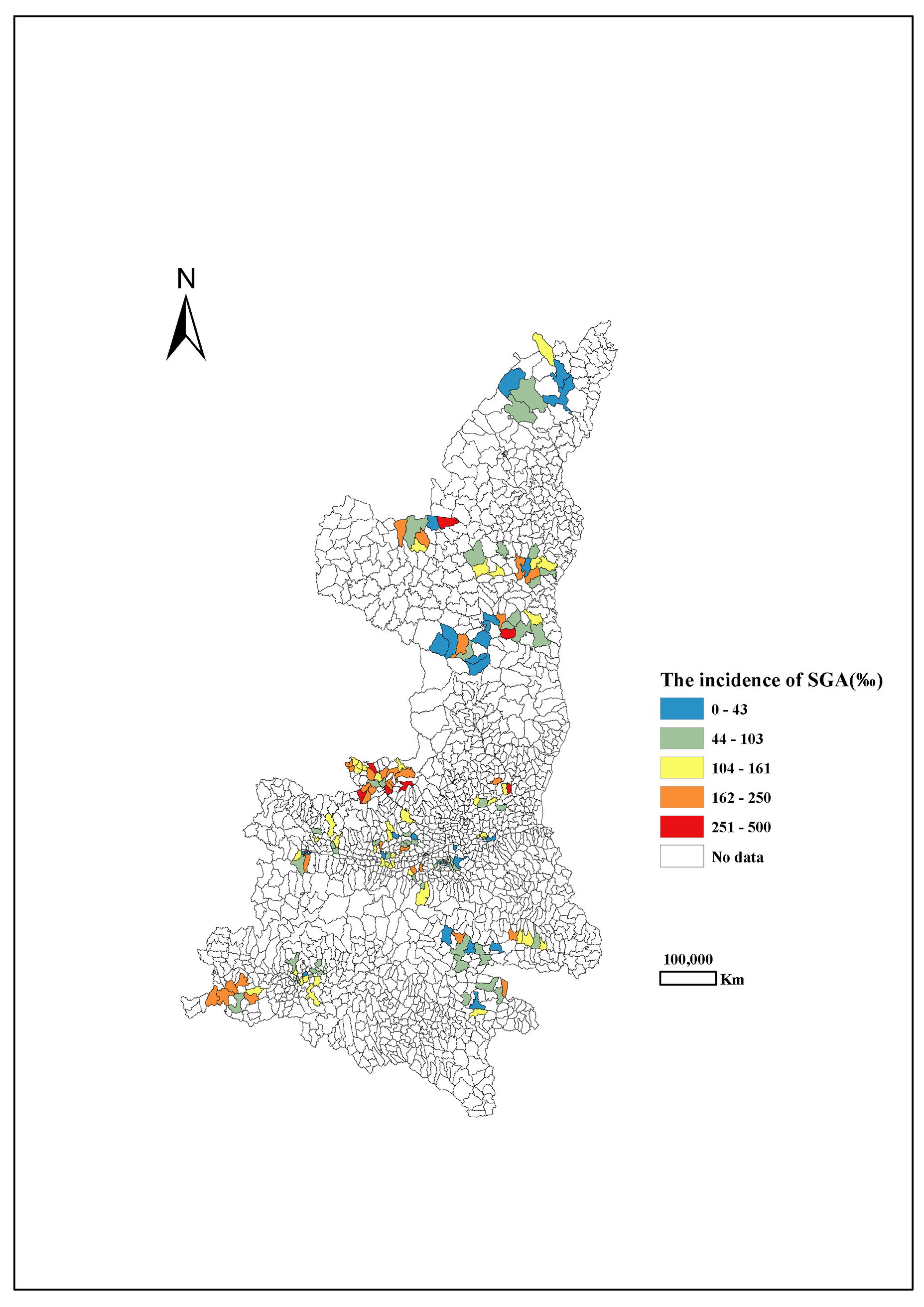

Supplement: S4 Fig — (TIF) [file pone.0254891.s009.tif]

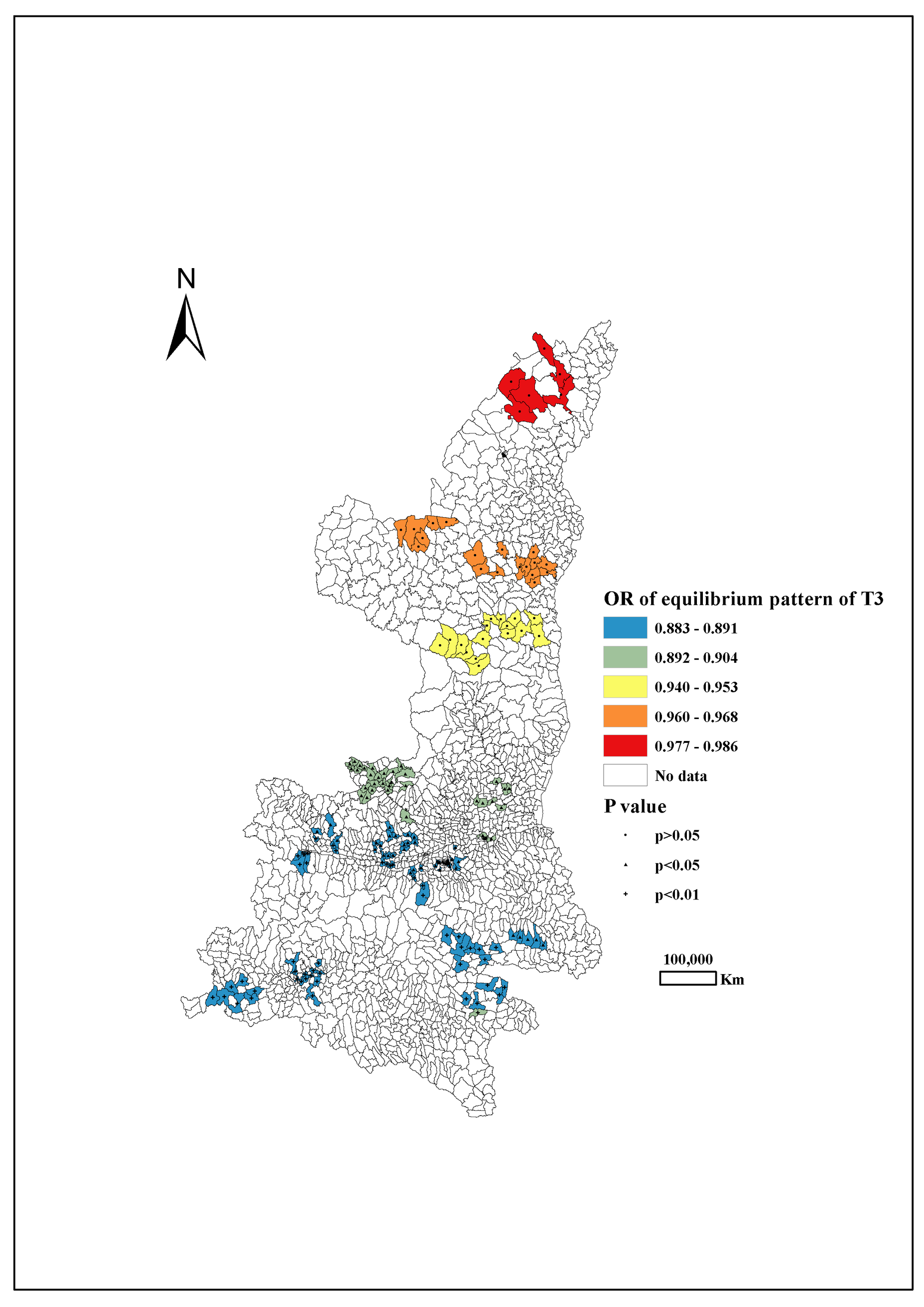

Supplement: S5 Fig — (TIF) [file pone.0254891.s010.tif]

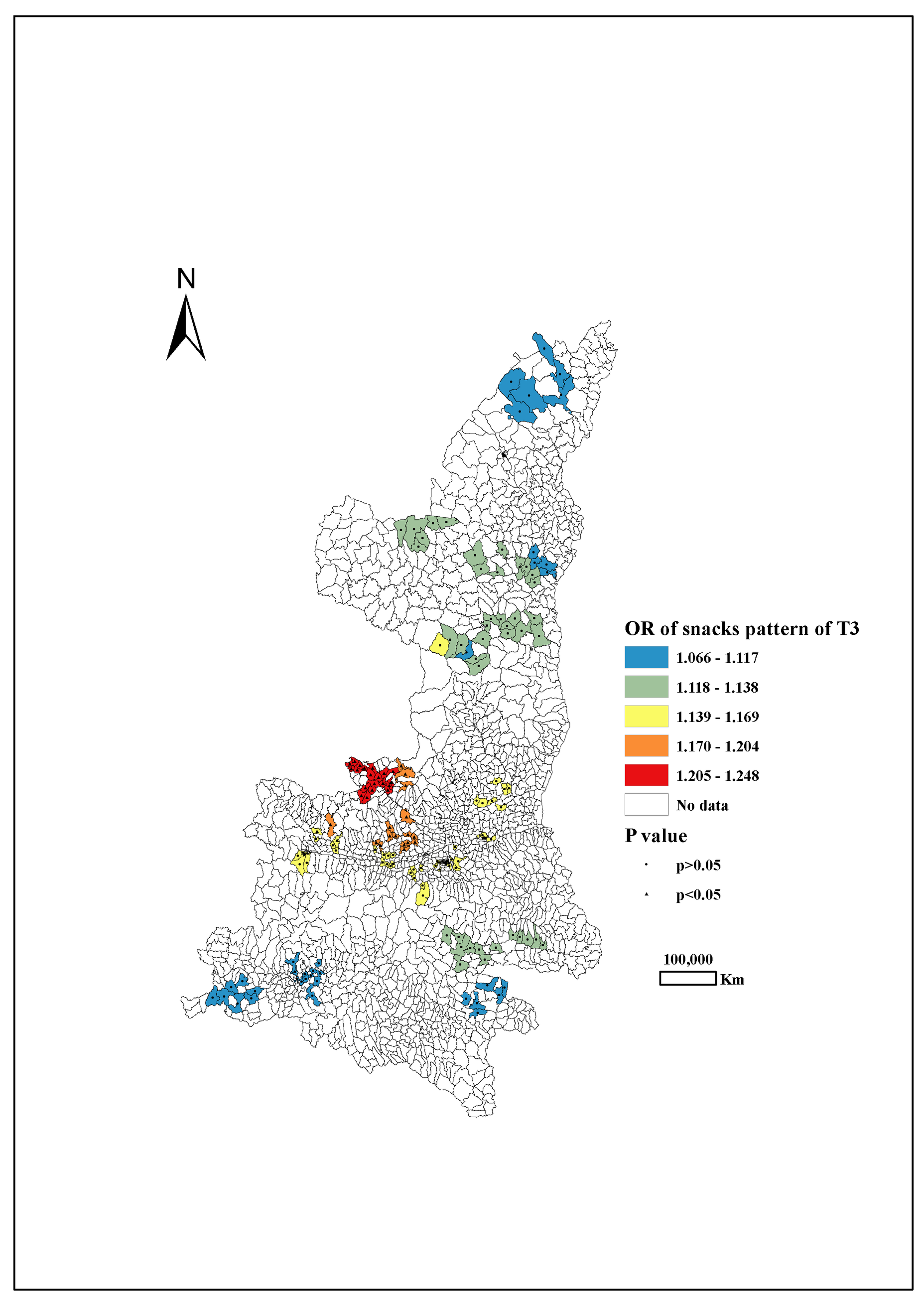

Supplement: S6 Fig — (TIF) [file pone.0254891.s011.tif]

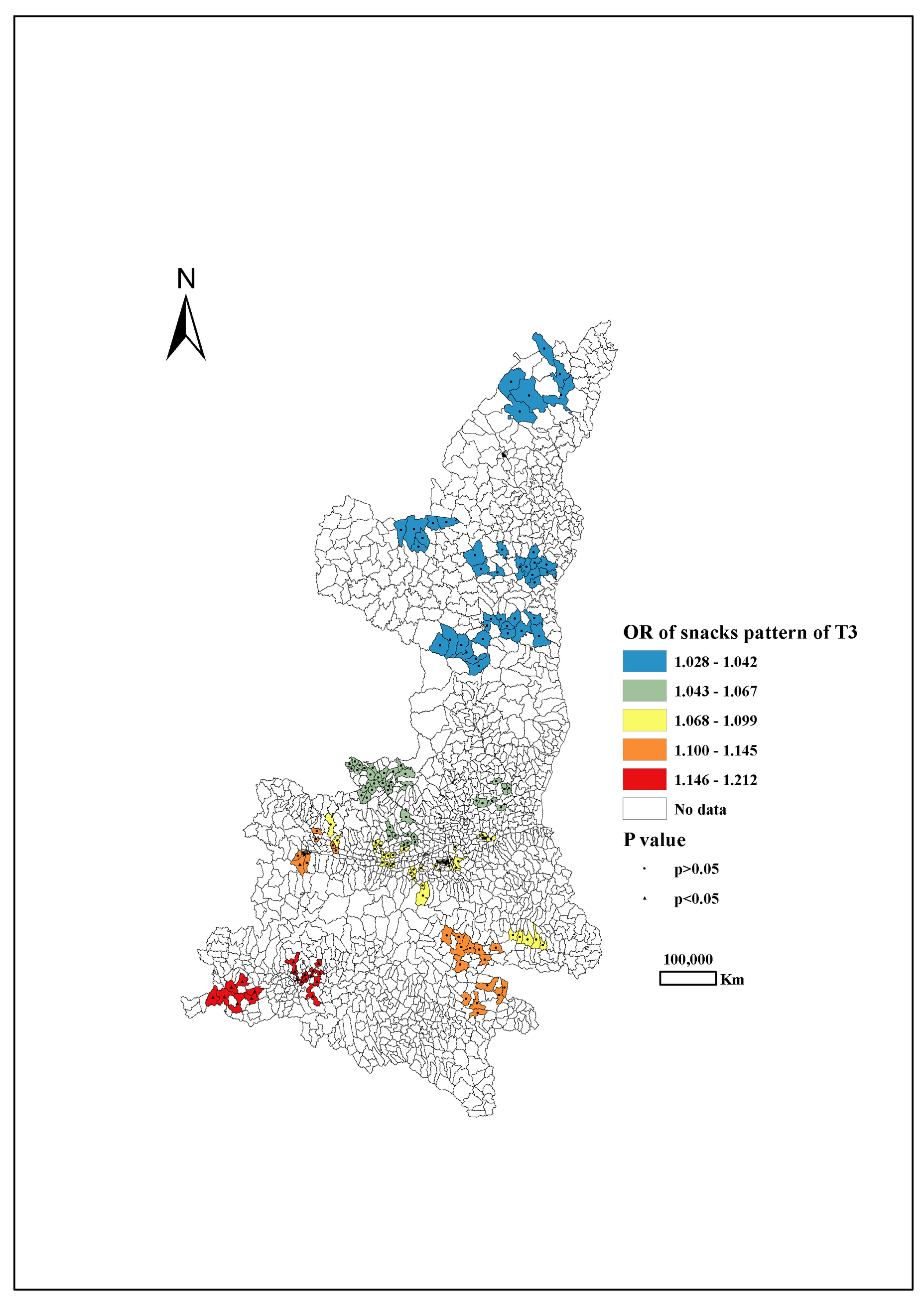

Supplement: S7 Fig — (TIF) [file pone.0254891.s012.tif]

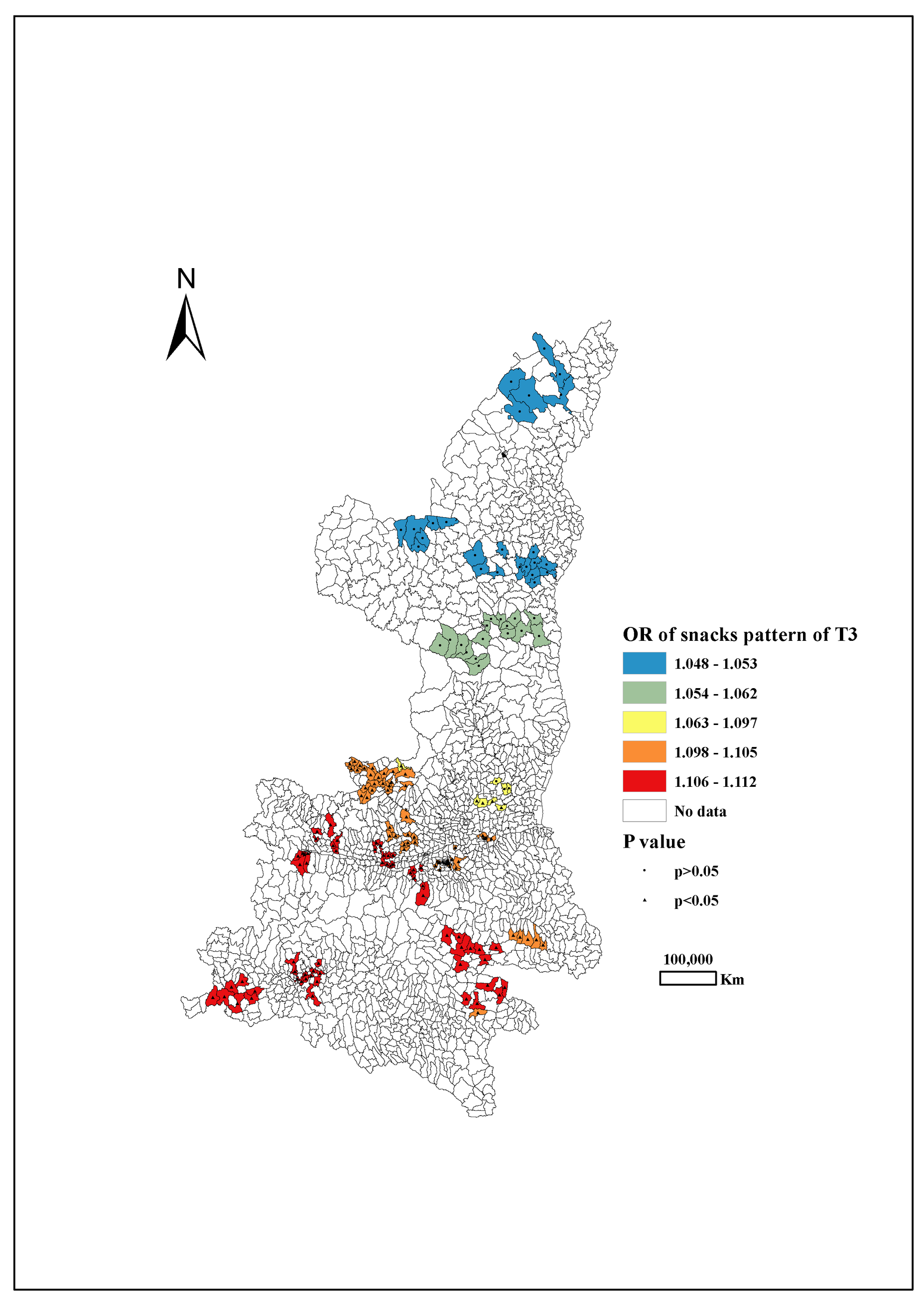

Supplement: S8 Fig — (TIF) [file pone.0254891.s013.tif]

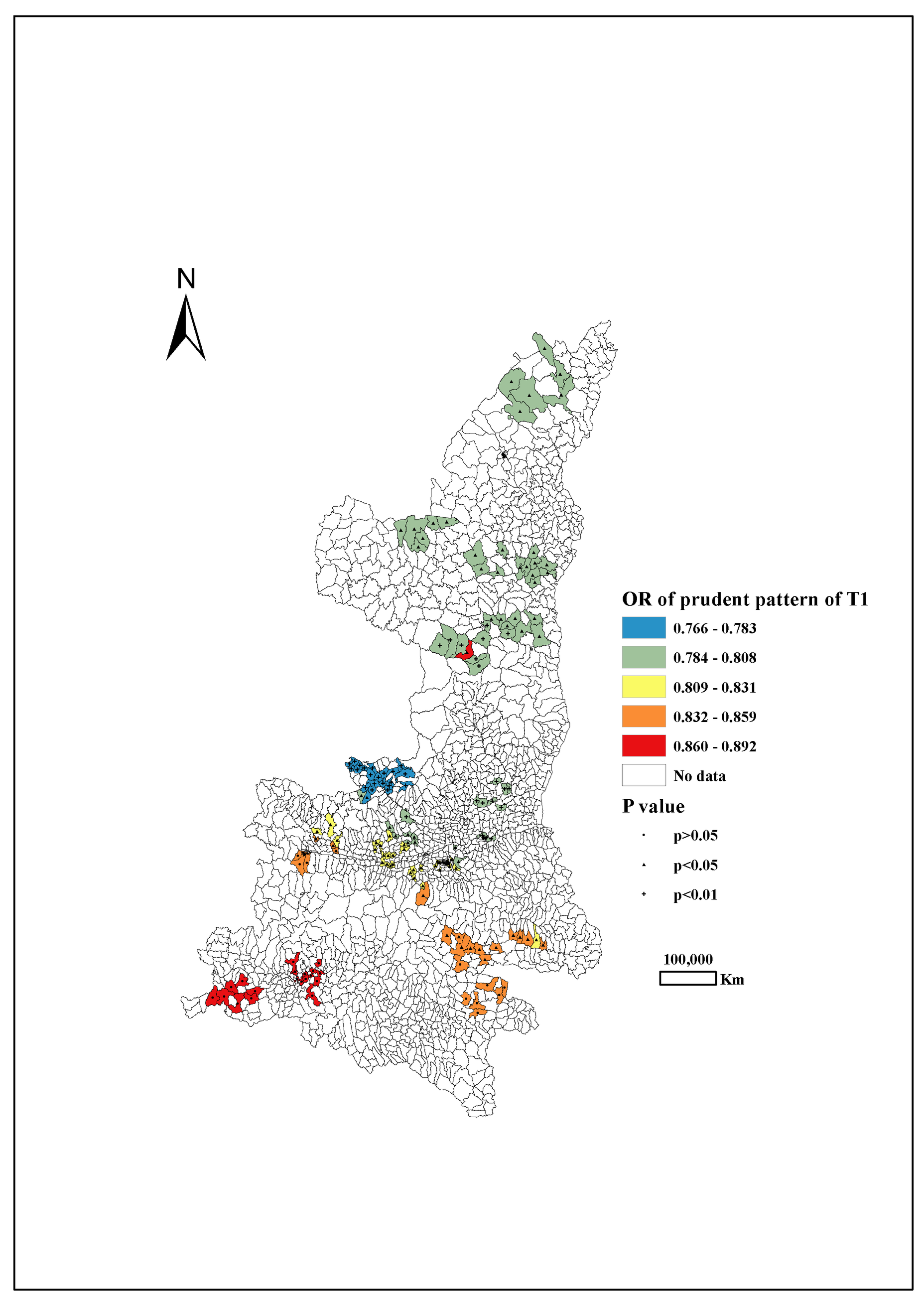

Supplement: S9 Fig — (TIF) [file pone.0254891.s014.tif]

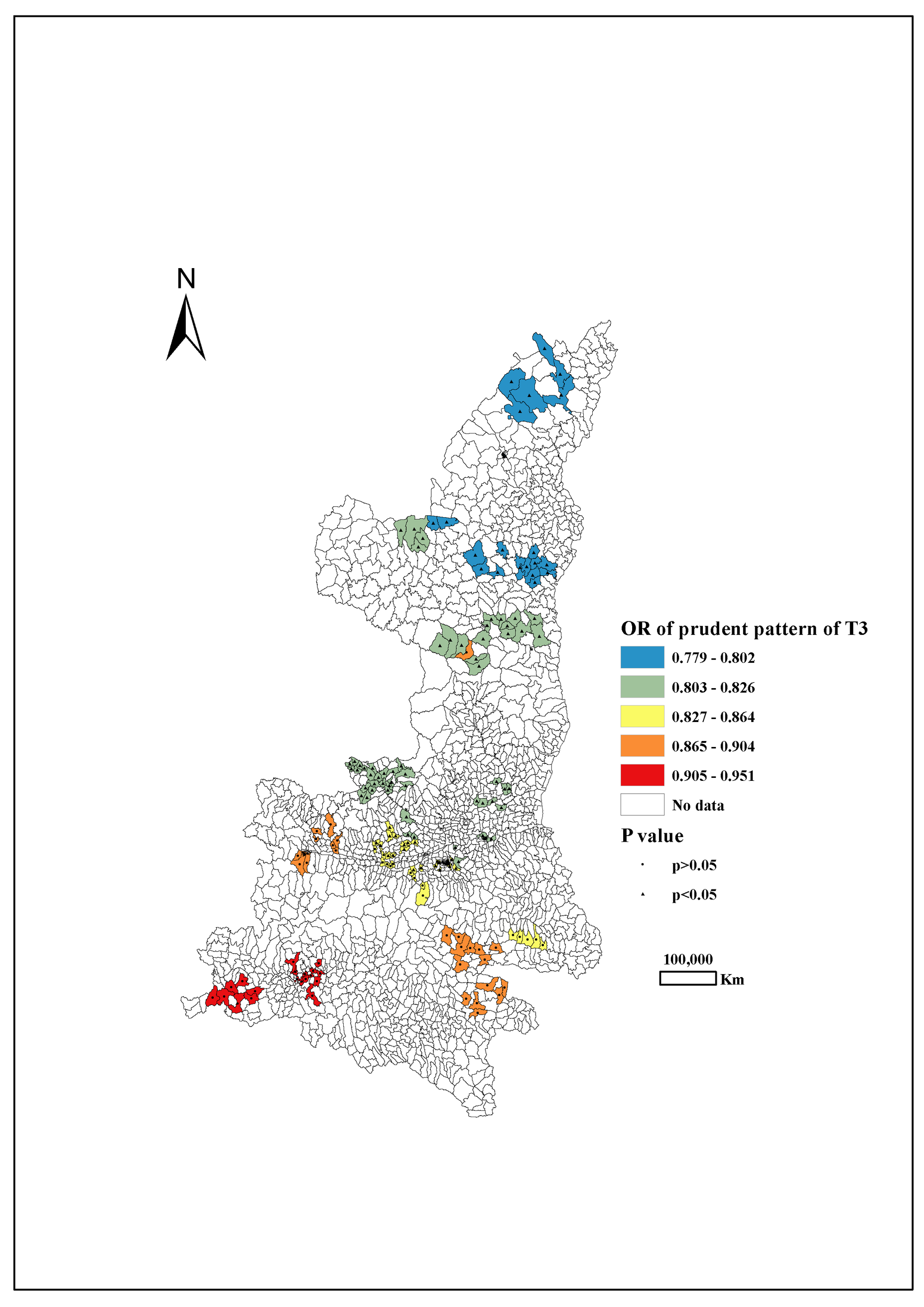

Supplement: S10 Fig — (TIF) [file pone.0254891.s015.tif]

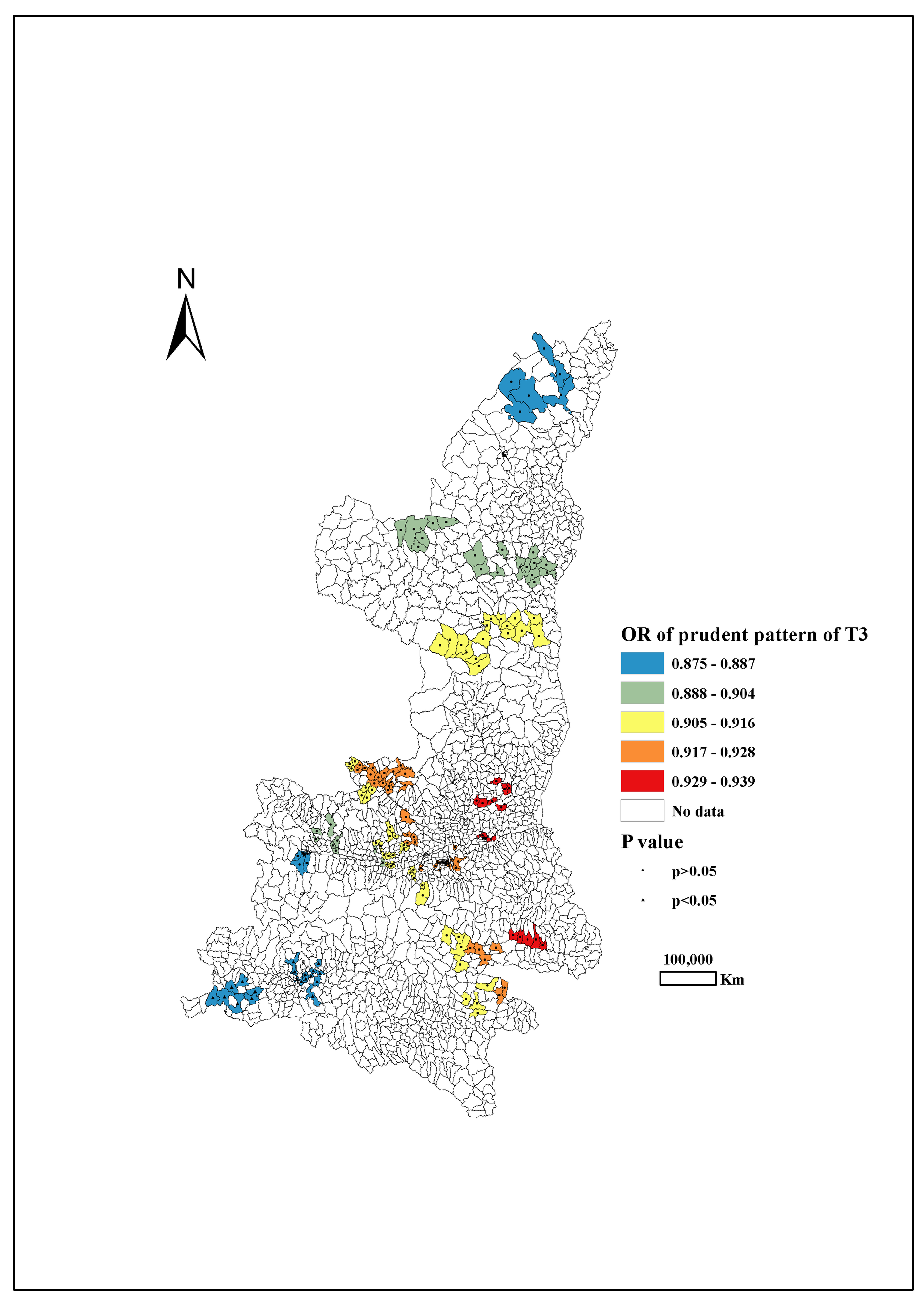

Supplement: S11 Fig — (TIF) [file pone.0254891.s016.tif]
