# Supplementary material for: Geographical variations in maternal dietary patterns during pregnancy associated with birth weight in Shaanxi province, Northwestern China
Source: PLoS One. 2021 Jul 22;16(7):e0254891. doi: 10.1371/journal.pone.0254891 (PMC8297908; doi:10.1371/journal.pone.0254891)
Supplement: Attachment — Submitted filename: PONE-D-20-40026-review_resume.pdf [file pone.0254891.s018.pdf]

COMMENT FOR THE AUTHOR

General comments . This manuscript was fascinating. However, it needs to add confirmation to give more information clearly.

A.1. Line 81. The survey was held from August to November 2013. Line 82, the objects were live birth born during 2010-2013. Kindly add specific time as inclusion criteria of this study to describe the study object more clearly e.g Baby born in January-August 2013 . Add in the method section.

A.2. Line 82. Material and methods. The diet survey questionnaire was used on the basis of the respondent's ability to recall history during pregnancy. Certainly, it has potential reminder bias.The subjects in this analysis are live baby mothers born in 2010-2013. It indicates different age ranges.

How does the author control the reminder bias based on different birth dates, e.g., the neonate mother (0-28 days) can remember better a dietary frequency than moms between 12-44 months of age? Perhaps you can explain the method of quality control.

A.3. Line 89. Why did the author make a different number of babies born as a topic in a rural and urban area? Ten villages and 20 communities? Depending on the number of children born in both areas? Has the author found a list of babies born in every county in urban or rural areas? Are the samples selected by the proportionate probability size (PPS) method? If yes. Please add in the process.If not, does the author use a weight to statistical analysis?

A.4. Line 130. Did the pattern equilibrium, snack, and prudent was derived from the explanatory factor/ confirmatory factor? Before taking PCA analysis? Add in the method. What software was used to doing PCA in this study? SPSS? STATA? Describe in this section.

A.5. If available, please identify geographical areas and add on the results section too? It is essential, the readers know about the classification, maybe most of the geographical kind, of three classified areas (northern, central, and southern), which is the coastal area? Mountainous? High land? For Urban, it is metropolitan, small-town Probably it related to different dietary trends

A.6. Line 175. Most of the respondents live in central province Shaanxi. Is the distribution of participant influence in the dietary pattern? Maybe potential to be modified factor. Please explain how the author took a sample from this large survey for this study purposes in the Methods section.

Why did the author consider using the whole data of a large survey? Did the author consider resampling to this analysis purposes so it can representative in three areas? Due to the aim study to comparing three area?

A.7. Is the author want to compare three areas? The author shows the distribution of dietary patterns based on geographical area, while the author also shows the participant proportion based on three areas is different (unbalanced). The various distribution proportions on the three areas potentially be a modifying factor. In table 2, we can see a significantly different dietary pattern between the three areas based on the X2 test. How the author control this issue?

A.8. Table 2. Kindly show the number of samples n (%) to shows balanced samples in each category.

A.9. Line 174 shows the different distribution of study participants. Has the author previously checked the various respondents' distribution in this area, probably influence this significance? Potential to be modified factor due to unbalanced distribution of samples, or consider reporting the effect in each region
(stratification) in multivariate if the study investigates the association between risk factor of birth outcome.

A.10. Table 2 and 3. For association purposes, the author can include adjusted p value from the multivariate analysis in this table with different mark, e.g ††

A.11. Line 220. We suggest not to use “varied in line 220“ because the proportion shows a slight difference.

A.12. Table 3. Refers to comment A10, to add p value multivariat analysis with different mark ††

A.13. Table 4. There is no socio-demographic characteristic was shown in this table, is only the dietary pattern that was a significant association with the birth outcome ? kindly add in the complete information in the previous table for socio demographics characteristic for multivariate test

A.14. General comment for table 5. How is it far different using nonspatial logistic regression and GWR?

A.15. Line 307 . Does it mean the different patterns mostly influenced by altitude? Why didn’t the author show others based on the rural and urban type of area

A.16. Line 354. Birth weight outcomes have close relation with the BMI of the mother (nutrition status of pregnant). Did the author consider this variable in their analysis or take in the limitation study.
