# Supplementary material for: Geographical variations in maternal dietary patterns during pregnancy associated with birth weight in Shaanxi province, Northwestern China
Source: PLoS One. 2021 Jul 22;16(7):e0254891. doi: 10.1371/journal.pone.0254891 (PMC8297908; doi:10.1371/journal.pone.0254891)
Supplement: Attachment — Submitted filename: PONE-D-20-40026_reviewer comments (1)-submit01022020.pdf [file pone.0254891.s019.pdf]

PLOS ONE

Geographical variations in maternal dietary patterns during pregnancy associated with birth weight in Shaanxi province, Northwestern China

--Manuscript Draft--

**Manuscript Number:**	PONE-D-20-40026
**Article Type:**	Research Article
**Full Title:**	Geographical variations in maternal dietary patterns during pregnancy associated with birth weight in Shaanxi province, Northwestern China
**Short Title:**	Spatial varying association of maternal dietary patterns in pregnancy with abnormal birth weight
**Corresponding Author:**	Leilei Pei, Ph.D.<br>School of Public Health and Institute of Public Health Research<br>Xi'an, Shaanxi, CHINA
**Keywords:**	birth weight (BW); maternal dietary patterns; low birth weight (LBW); macrosomia; small for gestational age (SGA); large for gestational age (LGA); spatial heterogeneity.
**Abstract:**	How to evaluate the geographical variation of maternal dietary patterns related to birth outcomes, are not known but are important for improving mothers and children health. Thus, the objective of the study was to investigate geographical variations of maternal dietary pattern during pregnancy, and evaluate the spatial varying association of maternal dietary patterns in pregnancy with abnormal birth weight. A population-based cross-sectional study was conducted in Shaanxi province in Northwest China in 2013 to evaluate the relationship between abnormal birth weight and dietary pattern using the Geographically Weighted Logistic Regression (GWLR). Three dietary patterns during pregnancy were extracted through factor analysis, explaining approximately 45.8% of the variability of food intake. Approximately 81.6% of mothers with higher scores on the equilibrium pattern was more unlikely to have a SGA infants, with the lower OR observed in Central and South Shaanxi. The snacks pattern was positively associated with LBW for 23.2% of participants, with the highest OR in Central Shaanxi. Among about 80.0% of participants with higher scores on the snacks pattern lived in South and Central Shaanxi, there was a higher risk for SGA. The OR values tend to descend from South to North Shaanxi. The OR values of the negative association between prudent pattern and LBW were observed to decrease from South to North Shaanxi, among approximately 59.3% of participants. The prudent pattern was also negatively associated with the increasing risk of fetal macrosomia among 19.2% of participants, mainly living in South Shaanxi. The association of maternal dietary patterns in pregnancy with abnormal birth weight, varied geographically across Shaanxi province. The findings emphasize the importance of geographical distribution to improve the dietary patterns among disadvantaged pregnant women.
**Order of Authors:**	Yini Liu
	Huihui Zhang
	Yaling Zhao
	Fangyao Chen
	Baibing Mi
	Jing Zhou
	Yulong Chen
	Duolao Wang
	Leilei Pei, Ph.D.
**Additional Information:**	
**Question**	**Response**

Powered by Editorial Manager® and ProduXion Manager® from Aries Systems Corporation

**Financial Disclosure**<br><br>Enter a financial disclosure statement that describes the sources of funding for the work included in this submission. Review the submission guidelines for detailed requirements. View published research articles from *PLOS ONE* for specific examples.<br><br>This statement is required for submission and **will appear in the published article** if the submission is accepted. Please make sure it is accurate.<br><br>**Unfunded studies**<br>Enter: *The author(s) received no specific funding for this work.*<br><br>**Funded studies**<br>Enter a statement with the following details:<br>• Initials of the authors who received each award<br>• Grant numbers awarded to each author<br>• The full name of each funder<br>• URL of each funder website<br>• Did the sponsors or funders play any role in the study design, data collection and analysis, decision to publish, or preparation of the manuscript?<br>• **NO** - Include this sentence at the end of your statement: *The funders had no role in study design, data collection and analysis, decision to publish, or preparation of the manuscript.*<br>• **YES** - Specify the role(s) played.<br><br>* typeset	The funders had no role in study design, data collection and analysis, decision to publish, or preparation of the manuscript.
**Competing Interests**<br><br>Use the instructions below to enter a competing interest statement for this submission. On behalf of all authors, disclose any competing interests that could be perceived to bias this work—acknowledging all financial support and any other relevant financial or non-financial competing interests.<br><br>This statement **will appear in the published article** if the submission is accepted. Please make sure it is	The authors have declared that no competing interests exist.

Powered by Editorial Manager® and ProduXion Manager® from Aries Systems Corporation

accurate. View published research articles from *PLOS ONE* for specific examples.<br><br>**NO authors have competing interests**<br><br>Enter: *The authors have declared that no competing interests exist.*<br><br>**Authors with competing interests**<br><br>Enter competing interest details beginning with this statement:<br><br>*I have read the journal's policy and the authors of this manuscript have the following competing interests: [insert competing interests here]*<br><br>* typeset	
**Ethics Statement**<br><br>Enter an ethics statement for this submission. This statement is required if the study involved:<br><br>• Human participants<br>• Human specimens or tissue<br>• Vertebrate animals or cephalopods<br>• Vertebrate embryos or tissues<br>• Field research<br><br>Write "N/A" if the submission does not require an ethics statement.<br><br>General guidance is provided below. Consult the submission guidelines for detailed instructions. **Make sure that all information entered here is included in the Methods section of the manuscript.**	N/A

**Format for specific study types**<br><br>**Human Subject Research (involving human participants and/or tissue)**<br>• Give the name of the institutional review board or ethics committee that approved the study<br>• Include the approval number and/or a statement indicating approval of this research<br>• Indicate the form of consent obtained (written/oral) or the reason that consent was not obtained (e.g. the data were analyzed anonymously)<br><br>**Animal Research (involving vertebrate animals, embryos or tissues)**<br>• Provide the name of the Institutional Animal Care and Use Committee (IACUC) or other relevant ethics board that reviewed the study protocol, and indicate whether they approved this research or granted a formal waiver of ethical approval<br>• Include an approval number if one was obtained<br>• If the study involved *non-human primates*, add *additional details* about animal welfare and steps taken to ameliorate suffering<br>• If anesthesia, euthanasia, or any kind of animal sacrifice is part of the study, include briefly which substances and/or methods were applied<br><br>**Field Research**<br><br>Include the following details if this study involves the collection of plant, animal, or other materials from a natural setting:<br>• Field permit number<br>• Name of the institution or relevant body that granted permission	
**Data Availability**<br><br>Authors are required to make all data underlying the findings described fully available, without restriction, and from the time of publication. PLOS allows rare exceptions to address legal and ethical concerns. See the PLOS Data Policy and FAQ for detailed information.	No - some restrictions will apply

A Data Availability Statement describing where the data can be found is required at submission. Your answers to this question constitute the Data Availability Statement and **will be published in the article**, if accepted.<br><br>**Important:** Stating 'data available on request from the author' is not sufficient. If your data are only available upon request, select 'No' for the first question and explain your exceptional situation in the text box.<br><br>Do the authors confirm that all data underlying the findings described in their manuscript are fully available without restriction?	
**Describe where the data may be found in full sentences. If you are copying our sample text, replace any instances of XXX with the appropriate details.**<br><br>• If the data are **held or will be held in a public repository**, include URLs, accession numbers or DOIs. If this information will only be available after acceptance, indicate this by ticking the box below. For example: *All XXX files are available from the XXX database (accession number(s) XXX, XXX.*).<br>• If the data are all contained **within the manuscript and/or Supporting Information files**, enter the following: *All relevant data are within the manuscript and its Supporting Information files.*<br>• If neither of these applies but you are able to provide **details of access elsewhere**, with or without limitations, please do so. For example:<br><br>*Data cannot be shared publicly because of [XXX]. Data are available from the XXX Institutional Data Access / Ethics Committee (contact via XXX) for researchers who meet the criteria for access to confidential data.*<br><br>*The data underlying the results presented in the study are available from (include the name of the third party*	All relevant data are within the manuscript and its Supporting Information files.

and contact information or URL).<br>• This text is appropriate if the data are owned by a third party and authors do not have permission to share the data.<br><br>* typeset	
Additional data availability information:	

Powered by Editorial Manager® and ProduXion Manager® from Aries Systems Corporation

Manuscript Click here to access/download;Manuscript;Manuscript.docx

Geographical variations in maternal dietary patterns during pregnancy associated with birth weight in Shaanxi province, Northwestern China

Yini Liu[1], Huihui Zhang[1], Yaling Zhao[1], Fangyao Chen[1], Baibing Mi[1], Jing Zhou[2], Yulong Chen[3], Duolao Wang[4], Leilei Pei[1]*

1 Department of Epidemiology and Health Statistics, School of Public Health, Xi'an Jiaotong University Health Science Center, Xi'an, Shaanxi 710061, P.R. China

2 Department of Pediatrics, The Second Affiliated Hospitical of Xi'an Jiaotong University, Xi'an, Shaanxi 710004, P.R. China

3 Institute of Basic and Translational Medicine, Shaanxi Key Laboratory of Ischemic Cardiovascular Disease, Shaanxi Key Laboratory of Brain Disorders, Xi'an Medical University, Xi'an, Shaanxi, 710021, P.R.China

4 Biostatistics Unit, Department of Clinical Sciences, Liverpool School of Tropical Medicine, Pembroke Place, Liverpool, L3 5QA, UK.

*Corresponding author: Leilei Pei, Department of Epidemiology and Health Statistics, School of Public Health, Xi'an Jiaotong University Health Science Center, Xi'an, Shaanxi 710061, P.R. China. Tel: +86 29 8265 5104. Fax: +86 29 8265 5387. Email: pll_paper@126.com

Abstract

How to evaluate the geographical variation of maternal dietary patterns related to birth outcomes, are not known but are important for improving mothers and children health. Thus, the objective of the study was to investigate geographical variations of maternal dietary pattern during pregnancy, and evaluate the spatial varying association of maternal dietary patterns in pregnancy with abnormal birth weight. A population-based cross-sectional study was conducted in Shaanxi province in Northwest China in 2013 to evaluate the relationship between abnormal birth weight and dietary pattern using the Geographically Weighted Logistic Regression (GWLR). Three dietary patterns during pregnancy were extracted through factor analysis, explaining approximately 45.8% of the variability of food intake. Approximately 81.6% of mothers with higher scores on the equilibrium pattern was more unlikely to have a SGA infants, with the lower OR observed in Central and South Shaanxi. The snacks pattern was positively associated with LBW for 23.2% of participants, with the highest OR in Central Shaanxi. Among about 80.0% of participants with higher scores on the snacks pattern lived in South and Central Shaanxi, there was a higher risk for SGA. The OR values tend to descend from South to North Shaanxi. The OR values of the negative association between prudent pattern and LBW were observed to decrease from South to North Shaanxi, among approximately 59.3% of participants. The prudent pattern was also negatively associated with the increasing risk of fetal macrosomia among 19.2% of participants, mainly living in South Shaanxi. The association of maternal dietary patterns in pregnancy with abnormal birth weight, varied geographically across Shaanxi province. The findings emphasize the importance of geographical distribution to improve the dietary patterns among disadvantaged pregnant women.

Introduction

Dietary patterns are useful to examine the quality of a person's overall diet related to health outcomes, because it is acknowledged that consumption of individual food or nutrients does not occur in isolation but interact with each other. Pregnancy is a critical period during which consuming a healthy diet characterized by a variety of nutritious foods is essential for promoting and maintaining maternal and child health [1,2,3,4]. Because of socio-cultural and geographical reasons, maternal dietary patterns in pregnancy is different and population-specific in each area [5,6]. Current researches showing association of maternal dietary patterns during pregnancy with adverse birth outcomes have not always been consistent across regions [7,8], which at least in part might have resulted from geographical heterogeneity. Spatial heterogeneity could cause biases for association estimates and their standard errors if they are not properly accounted for. Thus far, however, there was few evidence demonstrating the geographical difference in the relationship between maternal dietary patterns during pregnancy and birth outcomes of infants. Therefore, it is necessary to find an efficient way to uncover such spatially varying characteristics of maternal dietary patterns during pregnancy associated with birth outcomes, which would be helpful to better identifying links between diet and adverse birth outcomes.

Shaanxi located in Northwest China, is one of the provinces with huge variations in geographical environment and dietary structure in different areas. Thus, spatial heterogeneity cannot be ignored when we study the spatial variation of maternal dietary patterns during pregnancy in relation to birth outcomes. Abnormal birth weight (e.g. low BW or high BW), representing an important adverse birth outcome, is strongly correlated with various health outcomes, including neonatal mortality and morbidity as well as developing chronic diseases throughout their life [9,10,11,12]. In previous studies, however, conventional global regression models were commonly used to explore the association of maternal dietary pattern in pregnancy with abnormal birth weight. The traditional global regression, ignoring the impacts of local

variations, produce average parameters to reflect the spatial relationships equally over the whole study area. Due to disregarding the spatial heterogeneity, it is obvious that this evaluation is inadequate. For this issue, geographically weighted regression (GWR) model can be applied to explore how one dependent variable changes in response to one or more independent variables at the local scale. Through taking the samples within a defined neighbourhood into calculation by giving more weights to nearby samples than those further away, GWR generate local coefficients to elucidate the relationships within the neighbourhood. Using maps to show the geographical variation in the relative importance of each covariate, furthermore, GWR provides a visually powerful way of conveying the findings to public health practitioners.

In Shaanxi province of Northwest China, to the best of knowledge, how to evaluate the geographical variation of maternal dietary patterns related to birth outcomes, are not known but are important for improving mothers and children health. Thus, to address the aforementioned research questions, the objectives of this study were twofold: (1) to investigate geographical variations of maternal dietary pattern during pregnancy; (2) use GWR to evaluate the spatial varying association of maternal dietary patterns in pregnancy with abnormal birth weight.

Materials and Methods

Study design and participants

From August to November 2013, a large-scale cross-sectional survey with the purpose to assess the birth outcomes, was conducted in Shaanxi province, in Northwest China. The objects of the study were those live births born during 2010-2013 and their mothers, and participants were obtained by means of multi-stage random sampling method. In china, the administrative structure is divided into three layers, consisting of county, township and village in rural areas and district, street and community in urban areas, respectively. For the current study, 20 counties in rural areas and 10 districts in urban areas respectively, were randomly selected

Comment [A1]: Line 81. The survey was held from August to November 2013. Line 82, the objects were live birth born during 2010-2013. Kindly add specific time as inclusion criteria of this study to describe the study object more clearly e.g Baby born in January-August 2013 . Add in the method section.

Comment [A2]: Line 82. The diet survey questionnaire was used on the basis of the respondent's ability to recall history during pregnancy. Certainly, it has potential reminder bias.The subjects in this analysis are live baby mothers born in 2010-2013. It indicates different age ranges

How does the author control the reminder bias based on different birth dates, e.g., the neonate mother (0-28 days) can remember better a dietary frequency than moms between 12-44 months of age? Perhaps you can explain the method of quality control.

in the whole province. From each selected counties of rural areas, 6 villages each from 6 townships were randomly selected. In urban areas, however, from each selected districts, 6 communities from each of 3 selected streets were sampled randomly. Finally, 10 babies born during 2010-2013 and their mothers in each chosen villages and 20 in each chosen communities were selected using a completely random sampling method. A total of 7,934 eligible women who fulfilled completely the diet survey, were selected into the study. The study was conducted in accordance with the Helsinki Declaration and was approved and reviewed by the Ethics Committee of Xi'an Jiaotong University Health Science Center, (approval code: 2012008) [13].

Comment [A3]: Line 89. Why did the author make a different number of babies born as a topic in a rural and urban area? Ten villages and 20 communities? Depending on the number of children born in both areas? Has the author found a list of babies born in every county in urban or rural areas?

Are the samples selected by the proportionate probability size (PPS) method? If yes. Please add in the process.If not, does the author use a weight to statistical analysis?

Data collection

All information in the study were reported by mothers of the neonates using structured questionnaire through face-to-face interview. Firstly, a standard family questionnaire was adopted to collect socio-demographic characteristics. Secondly, a semi-quantitative food frequency questionnaire (FFQ) was used to assess dietary intakes of mothers during the whole pregnancy. The FFQ in the survey has been previously validated to satisfied reliability and validity for pregnant women in rural western China [14,15]. A total of 102 food items were included in the FFQ, covering fourteen food groups (cereals, potatoes, legumes, vegetables, fungi, fruits, nuts, meats, dairy, eggs, fish, ethnic snacks, fast foods, and beverages) based on the China Food Composition Table [16]. The frequency of 102 food items was divided into eight categories, ranging from "never" to "≥ 2 times/day". With the help of food portion images, the portion sizes for the 102 food items were recorded [14,15]. The information of neonates, including birth weight, birth date, and so on, was recorded from medical records.

Quality control

Field surveys and data collection were carried out by the trained field staff from Xi’an Jiaotong University Health Science Center. Prior to the formal survey, all questionnaires and procedures were pretested in field,

and then detailed interview guidelines were developed. Ten investigation teams from Xi’an Jiaotong University Health Science Center were established for these surveyed counties or districts, and each was composed of 10-12 qualified investigators, a supervisor from Xi'an Jiaotong University Health Science Center, and a pediatrician from the local maternal and child health hospital. During the field survey, the supervisors were required to monitor all field work and review the questionnaire. As soon as any errors and missing items were found, participants were re-interviewed immediately. With sustained support of local hospitals, health administration departments and the Provincial Ministry of Health, all information was collected in local village clinics and community health service centers [13].

Study variables

Abnormal birth weight was considered final outcome variables in the multivariate analysis: including low birth weight (LBW, birth weight < 2500 g), macrosomia (birth weight > 4000 g), small for gestational age (SGA, birth weight was below the 10th percentile of the average gestational age) and large for gestational age (LGA, birth weight was above the 90th percentile of the average gestational age) [17,18]. Dietary pattern as the independent variable had two dummy variables, respectively: T1 (1 = yes, 0 = no), T3 (1 = yes, 0 = no), with T2 as a reference group. The socio-demographic variables were as follows: child gender(male, female), fetal number (singleton, twin and multi-fetal), infant parity (1, 2, or ≥ 3), childbearing age (18-24, 25-29, ≥ 30 years), mother’s education (primary school and below, junior high school, senior high school, college and above), mother's residence during pregnancy (floating, permanent), Household wealth Index (poor, middle-income, rich), altitude of residence (< 500, 500-1000, ≥ 1000), and area (South Shaanxi, Central Shaanxi, North Shaanxi).

Statistical analysis

A database was established using Epidata 3.1 software by double entry. Maternal dietary patterns were derived

by principal component analysis (PCA) and varimax rotation method. Before identifying the dietary patterns, the reliability of factor analysis was tested based on the Kaiser–Meyer–Olkin value and Bartlett's test of sphericity. The number of dietary pattern was determined by eigenvalues >1.2, variance contribution rate (>5%), the scree plot and the interpretability of the factors [19,20]. It was considered that food groups with absolute factor loadings >0.3 were highly related to the identified factors. Factor scores were calculated for each mother, indicating the extent to which mother's diet cohered to the respective dietary patterns.

Comment [A4]: Line 130. Did the pattern equilibrium, snack, and prudent was derived from the explanatory factor/ confirmatory factor? Before taking PCA analysis? Add in the method.

What software was used to doing PCA in this study? SPSS? STATA? Describe in this section.

The geographic information (including geographic coordinates and altitude) for each township/street in Shaanxi province, was obtained from the vector map of China through the Geographic Information Comprehensive Service Website (http://www.tianditu.com/). The basic geographic unit in the study was townships and streets, of which the geographic location was defined as the geographic coordinates (ie latitude/longitude) of the township/street center where the participants lives.

The GWLR model was employed to evaluate the relationship between abnormal birth weight and individual factors, and the formula is as follows [21]:

$$\log\left(\frac{P(y_i = 1)}{1 - P(y_i = 1)}\right) = \beta_0(u_i, v_i) + \sum_{j=1}^{k} \left(\beta_j(u_i, v_i)x_{ij}\right)$$

The equation hypothesize that y_i (dependent variables) was any abnormal birth weight for each individual i, x_{ij} represented a set of independent variables $(j = 1, \dots, k)$ for individual i, (u_i, v_i) was regarded as the x-y coordinates of individual i; β_{ij} (regression coefficient) was deemed as the estimated effect of independent variable j for individual i. The GWLR model is actually the extension and deformation of the ordinary linear regression model. The spatial data was incorporated into the model, to create conditions for analyzing the spatial characteristics of the regression relationship.

We selected the GWR 4.0 software in this research

(https://geodacenter.asu.edu/software/downloads/gwr_downloads), the GWLR model was estimated by the iterative reweighted least squares method. During the process of modeling, a distance-based weighting scheme was used to assign weights to each township/street and estimated the GWLR equation for each township/street. The kernel type and function for geographic weighting to estimate local coefficients for each township/street and bandwidth size was adaptive bisquare. In order to select bandwidth of the adaptive kernel, the Corrected Akaike Information Criterion (AICc) was adopted as the golden section search method. The variability of each coefficient in geographic space was tested by comparing different [22]. In the GWLR model, we obtained the OR corresponding to the unit change in the variable by exponentiation of the explanatory variable coefficients [22]. We attempted to evaluate the variability of the influence of abnormal birth weight on individuals in different regions by comparing the variability of each coefficient in geographic space [23].

The complete analysis is as follows. Firstly, non-spatial logistic regression analysis was performed using IBM SPSS Statistics 25.0, to estimate the effects of each dietary pattern on abnormal birth weight. For bilateral test, the test level was $\alpha=0.05$. Afterward, all explanatory variables were entered into the GWLR model, as mentioned above. The variables with the largest p-value were gradually removed from the model until only those with local p-value <0.1 were retained. Eventually, ArcGIS 10.0 software (Environmental Systems Research Institute, Inc., Redlands, CA, US) was used to display the local average estimates (OR) and p-values of individuals on the Shaanxi map, to intuitively reflect the geographical differences in the relationship between abnormal birth weight outcome and individual factors.

Results

Baseline characteristics of the participants

In this study, a total of 173 town/street were chosen within 30 counties in Shaanxi province, and 7,934 live infants were included in the final analysis. Among the mothers included in the study, average age was

Comment [A5]: If available, please identify geographical areas and add on the results section too? It is essential, the readers know about the classification, maybe most of the geographical kind, of three classified regions (northern, central, and southern), which is the coastal area? Mountainous? High land? For Urban, it is metropolitan, small-town Probably it related to different dietary trends

26.88±4.62 years; average parity was 1.44 ± 0.56. The proportions of study participants from northern, central, and southern Shaanxi, were 18.1, 51.1 and 30.7%, respectively. Of the infants, the average age was 8.92 ±6.30 months (range 0-45 months); 53.6% were male; 98.8% were singletons.

Comment [A6]: Line 175. Most of the respondents live in central province Shaanxi. Is the distribution of participant influence in the dietary pattern? Maybe potential to be modified factor.

Please explain how the author took a sample from this large survey for this study purposes in the Methods section.

Why did the author consider using the whole data of a large survey? Did the author consider resampling to this analysis purposes so it can representative in three regions? Due to the aim study to comparing three area?

Characteristics of dietary pattern distribution during pregnancy among mothers

Through principal component analysis, three dietary patterns during pregnancy were extracted in Shaanxi province, northwest China, which explained approximately 45.8% of the variability in food intake (28.4%, 9.5% and 7.9% for three dietary patterns, respectively). According to the high positive loadings from various foods, including vegetables, homonemeae, beans meats, meat or flesh, fruit, fish and shrimp, potato, nuts, dairy, fast-food, sweetmeat, cereal, eggs and beverages, this first pattern was labelled the 'equilibrium pattern'. The scores of the equilibrium pattern were highest in Central Shaanxi and lowest in Northern Shaanxi. (Fig 1). The second pattern labelled 'snacks pattern', had high loading from beverages, sweetmeat, fast-food, dairy and eggs. The scores of the snacks pattern were higher in North Shaanxi, followed by South Shaanxi and Central Shaanxi (Fig 2). The third pattern with high intakes of dairy products and eggs, was labelled the 'prudent pattern' (Table 1). It appeared a decreasing trend of the scores of the prudent pattern from south to north. (Fig 3).

Comment [A7]: Is the author want to compare three regions? The author shows the distribution of dietary patterns based on geographical area, while the author also shows the participant proportion based on three areas is different (unbalanced). The various distribution proportions on the three regions potentially be a modifying factor. In table 2, we can see a significantly different dietary pattern between the three areas based on the X2 test. How the author control this issue?

Fig 1. The score of equilibrium pattern among pregnant women by different areas in Shaanxi province, 2013.

Fig 2. The score of snacks pattern among pregnant women by different areas in Shaanxi province, 2013.

Fig 3. The score of prudent pattern among pregnant women by different areas in Shaanxi province, 2013.

Table 1. Factor loadings for the identified dietary patterns among pregnant women (n 7934) in

Shaanxi province, Northwest China, 2013

Dietary pattern	Food group	Factor loading*	Cumulative variance explained (%)
Equilibrium pattern	vegetable	0.762	28.4
	homonemeae	0.695	
	beans	0.624	
	meat or flesh	0.602	
	fruit	0.590	
	fish and shrimp	0.537	
	potato	0.535	
	nuts	0.504	
	dairy	0.469	
	fast-food	0.469	
	sweetmeat	0.443	
	cereal	0.357	
	eggs	0.338	
	beverages	0.315	
Snacks pattern	beverages	0.641	37.9
	sweetmeat	0.574	
	fast-food	0.348	
	dairy	-0.333	
	eggs	-0.437	
Prudent pattern	eggs	0.408	45.8
	dairy	0.331	
	vegetable	-0.342	
	potato	-0.534	

*Food groups are listed by the size of factor loadings, with negative loadings at the end. Food groups with absolute factor loadings <0.300 are not shown.

Table 2 displayed sociodemographic characteristics and median daily intakes of energy and nutrients according to tertiles of dietary patterns scores among pregnant women in Shaanxi province. In terms of equilibrium pattern, pregnant women with high scores were more likely to live in south or central Shaanxi, lower altitudes areas (<500 meters), be better educated and wealthier. Besides, there were higher childbearing age and lower parity among mothers with high scores on equilibrium pattern. The sociodemographic characteristics of the prudent pattern was similar to the equilibrium pattern, except for maternal childbearing age which didn’t differ between tertiles of prudent pattern scores. On the contrary, those with high scores on

the snacks pattern were more likely to live in north Shaanxi, higher altitudes areas (>1000 meters), be less educated and poor, and be aged 18-24 years at delivery. Women with higher scores on the equilibrium pattern had higher intakes of energy and all selected nutrients, while those with lower scores on the prudent pattern and the snacks pattern had higher energy intake, carbohydrate intake and higher intakes of other selected nutrients.

Table 2. The characteristics of dietary patterns among pregnant women (n 7934) in Shaanxi province, Northwest China, 2013

Comment [A8]: Table 2. Kindly show the number of samples n (%) to shows balanced samples in each category.

	**Equilibrium pattern**				**Snacks pattern**				**Prudent pattern**			
	**T1**	**T2**	**T3**	**P***	**T1**	**T2**	**T3**	**P***	**T1**	**T2**	**T3**	**P***
Sociodemographic characteristics†												
Child gender (%)				0.490				0.953				0.244
Male	32.7	33.5	33.8		33.4	33.3	33.2		34.0	32.6	33.5	
Female	34.0	33.1	32.9		33.2	33.3	33.5		32.5	34.2	33.3	
Fetal number (%)				0.051				0.746				0.809
Singleton	33.4	33.2	33.5		33.3	33.3	33.4		33.3	33.3	33.4	
Twin and multi-fetal	29.2	44.8	26.0		33.3	36.5	30.2		31.3	32.3	36.5	
Infant parity (%)				<0.001				<0.001				<0.001
1	30.8	32.7	36.5		36.7	31.2	32.1		29.8	32.7	37.6	
2	37.1	34.1	28.9		28.7	36.2	35.1		37.6	34.7	27.7	
≥3	37.1	36.3	26.6		27.8	37.8	34.4		49.4	27.0	23.6	
Childbearing age (%)				<0.001				<0.001				0.201
18-24	36.3	32.5	31.2		28.5	31.6	39.9		33.7	34.0	32.3	
25-29	30.4	34.7	35.0		36.2	33.5	30.3		32.3	33.8	33.9	
≥30	34.3	32.0	33.8		35.3	35.4	29.3		34.7	31.4	33.9	
Mother’s education (%)				<0.001				<0.001				<0.001
Primary school and below	42.1	35.4	22.5		21.2	37.6	41.2		43.7	34.4	21.9	
Junior high school	37.3	33.4	29.4		28.4	35.0	36.6		35.8	34.4	29.8	
Senior high school	29.5	32.6	37.9		37.5	32.3	30.2		28.8	33.6	37.6	
College and above	19.0	33.1	47.9		51.9	26.2	21.8		24.8	28.3	46.9	
Mother's residence during pregnancy (%)				0.141				0.001				<0.001
Floating	30.7	33.6	35.7		38.0	32.7	29.2		26.7	32.4	40.9	

Permanent	33.6	33.3	33.1		32.7	33.4	34.0		34.3	33.3	32.4	
Household wealth Index (%)				<0.001				<0.001				<0.001
Poor	36.9	35.9	27.2		29.5	35.5	35.0		37.7	34.4	27.8	
Middle-income	32.2	33.1	34.7		34.7	33.3	32.0		33.5	32.8	33.7	
Rich	31.0	31.0	38.0		35.5	31.3	33.2		29.0	32.7	38.3	
Altitude (%)				<0.001				<0.001				<0.001
<500	28.3	31.9	39.8		42.6	30.4	27.0		22.9	32.4	44.7	
500-1000	34.8	32.0	33.2		31.5	34.4	34.1		31.0	33.7	35.3	
>1000	36.5	37.5	26.0		25.3	34.9	39.8		50.3	33.5	16.2	
Area (%)				<0.001				<0.001				<0.001
South area	35.4	29.4	35.3		30.0	32.1	37.9		25.5	32.8	41.7	
Central area	31.8	33.2	35.1		40.0	34.1	25.9		29.4	35.3	35.3	
North area	34.1	40.3	25.6		19.9	33.1	47.0		57.4	28.6	14.0	
Median daily intake												
Energy(KJ)	4417	6681	10120	<0.001	8329	5933	6011	<0.001	7375	5930	6925	<0.001
Protein(g)	39	61	97	<0.001	82	53	49	<0.001	65	53	66	<0.001
Fat(g)	20	38	69	<0.001	54	31	30	<0.001	35	32	48	<0.001
Carbohydrate(g)	195	276	388	<0.001	320	254	260	<0.001	325	251	255	<0.001
Vitamin A(μg RE)	169	392	794	<0.001	500	325	387	<0.001	523	306	380	<0.001
Thiamin(mg)	0.4	0.6	1	<0.001	0.8	0.6	0.6	<0.001	0.8	0.6	0.6	<0.001
Riboflavin(mg)	0.3	0.7	1.2	<0.001	0.9	0.5	0.5	<0.001	0.7	0.5	0.8	<0.001
Folate(μg)	141	273	510	<0.001	369	230	238	<0.001	315	225	293	<0.001
Niacin(mg)	7	11	18	<0.001	14	10	10	<0.001	13	10	11	<0.001
Vitamin C(mg)	42	89	166	<0.001	97	73	98	<0.001	120	73	77	<0.001
Vitamin E(mg)	6.9	16.6	31	<0.001	22	14	14	<0.001	19	14	17	<0.001
Ca(mg)	235	462	795	<0.001	666	392	362	<0.001	470	392	545	<0.001
Fe(mg)	8.3	14.6	25.8	<0.001	20	13	13	<0.001	17	13	16	<0.001

Comment [A9]: Line 174 shows the different distribution of study participants. Has the author previously checked the various respondents' distribution in this area, probably influence this significance? Potential to be modified factor due to unbalanced distribution of samples, or consider reporting the effect in each region (stratification) in multivariate if the study investigates the association between risk factor of birth outcome.

Zn(mg)	2.8	5.3	9.6	<0.001	7.3	4.4	4.6	<0.001	5.8	4.5	6.1	<0.001
Se(μg)	17	28	49	<0.001	40	24	22	<0.001	27	24	34	<0.001

T, tertiles; RE, retinol equivalent.

*P values for the differences among the groups of dietary pattern scores were derived from the $\chi 2$ test for categorical variables and the Kruskal–Wallis test for continuous variables.

Comment [A10]: Table 2 and 3. For association purposes, the author can include adjusted p value from the multivariate analysis in this table with different mark, e.g ††

†Values are presented as percentages for these categorical variables.

Characteristics of abnormal birth weight

Among the live infants, the prevalence of abnormal birth weight was 3.7% for LBW, 6.2% for macrosomia, 10.9% for SGA, 10.0% for LGA, respectively. The prevalence of LBW and LGA were the highest in South Shaanxi (4.1% for LBW and 10.3% for SGA, respectively), followed by Central Shaanxi (3.7% and 10.2%) and North Shaanxi (3.3% and 10.1%) (S1 and S2 Figs). The incidence of macrosomia also varied among Shaanxi province, the highest of which is in North Shaanxi (6.6%), followed by Central Shaanxi (6.2%), Southern Shaanxi (6.1%) (S3 Fig). Our survey data indicated that SGA incidence was higher in Central Shaanxi (11.7%) than in South Shaanxi (10.9%) or Northern Shaanxi (10.0%). (S4 Fig). The prevalence of abnormal birth weight differed across the baseline characteristics, and are presented in Table 3. The incidence of LBW was lower in T3 group of equilibrium pattern, T1 group of snacks pattern, and T1 or T3 group of prudent pattern, respectively (P <0.05). Likewise, there were lower prevalence of SGA in T3 group of equilibrium pattern and T1 group of snacks pattern (P <0.05).

Comment [A11]:

Line 220. We suggest not to use "varied in line 220" because the proportion shows a slight difference.

Table 3. Baseline characteristics of study population (n 7934) by different areas in Shaanxi province, Northwest China, 2013*

Study variable	LBW	Macrosomia	SGA	LGA
	n (%)	n (%)	n (%)	n (%)
Child gender				
Male	139(3.3)	316(7.5)	461(11.0)	436(10.4)

Female	157(4.3)†	174(4.8)†	407(11.3)	358(9.9)
Fetal number				
Singleton	251(3.2)	488(6.3)	827(10.7)	786(10.2)
Twin and multi-fetal	45(46.9)†	2(2.1)	41(43.2)†	8(8.4)
Infant parity				
1	148(3.2)	251(5.4)	500(11.0)	414(9.1)
2	120(4.1)	220(7.5)	318(10.9)	350(11.9)
≥3	26(10.3)†	18(7.1)†	42(16.7)†	28(11.1)†
Childbearing age				
18-24	95(3.5)	134(5.0)	336(12.6)	216(8.1)
25-29	126(3.9)	200(6.2)	328(10.3)	324(10.1)
≥30	74(3.9)	152(8.1)†	200(10.8)†	243(13.1)†
Mother's education				
Primary school and below	27(3.8)	46(6.4)	92(13.0)	67(9.4)
Junior high school	173(4.1)	242(5.7)	503(12.0)	416(9.9)
Senior high school	71(4.1)	110(6.3)	185(10.8)	165(9.6)
College and above	25(2.1)†	91(7.7)	87(7.5)†	142(12.3)
Mother's residence during pregnancy				
Floating	43(3.7)	64(6.2)	106(11.2)	111(10.1)
Permanent	252(4.3)	423(6.4)	755(10.7)	678(11.2)
Household wealth Index				
Poor	104(4.1)	133(5.2)	329(13.0)	221(8.8)
Middle-income	89(3.4)	167(6.4)	274(10.6)	264(10.2)
Rich	103(3.8)	190(7.0)†	265(9.9)†	309(11.5)†
Altitude				
<500	74(3.2)	164(7.0)	198(8.6)	250(10.8)
500-1000	142(3.9)	221(6.1)	396(11.1)	372(10.5)
>1000	80(4.1)	105(5.4)	274(14.3)†	172(9.0)
Area				
South area	100(4.1)	148(6.1)	258(10.9)	244(10.3)
Central area	148(3.7)	248(6.2)	467(11.7)	406(10.2)
North area	48(3.3)	94(6.6)	143(10.0)	144(10.1)
Equilibrium pattern				
T1	116(4.4)	163(6.2)	310(12.0)	247(9.5)
T2	102(3.9)	148(5.6)	312(12.0)	262(10.1)
T3	78(3.0)†	179(6.8)	246(9.5)†	285(11.0)
Snacks pattern				
T1	76(2.9)	171(6.5)	254(9.8)	289(11.1)
T2	105(4.0)	143(5.4)	284(10.9)	241(9.3)
T3	115(4.4)†	176(6.7)	330(12.7)†	264(10.2)
Prudent pattern				
T1	87(3.3)	157(6.0)	296(11.4)	247(9.5)

T2	121(4.6)	176(6.7)	299(11.5)	277(10.7)
T3	88(3.3)†	157(6.0)	273(10.5)	270(10.4)

T, tertiles; LBW, low birth weight; SGA, small for gestational age; LGA, large for gestational age.

* Values are the number of abnormal birth outcomes and the prevalence is included in the bracket.

† Significant differences among the groups were derived from the χ2 test for categorical variables (P<0·05).

Comment [A12]: Table 3. Refers to comment A10, to add p value multivariat analysis with different mark
††

Association between dietary patterns and abnormal birth weight using non-spatial logistic regression

According to the non-spatial logistic regression results (Table 4), SGA was less likely to occur among the infants born to mothers in T3 group of equilibrium pattern in comparison with those whose mothers were from T2 group (OR: 0.801, 95%CI: 0.665-0.965). The higher scores of snacks pattern was associated with the increasing risk for macrosomia and SGA among the infants (OR: 1.265, 95%CI: 1.000-1.602; OR: 1.260, 95%CI: 1.056-1.505, respectively). Compared with those in the T2 group of prudent pattern, mothers in the T1 group of the prudent pattern had higher risk for LBW in their offspring (OR: 0.732, 95% CI: 0.537-0.997), and mothers in the T3 group had a lower risk for fetal macrosomia (OR: 0.788, 95% CI: 0.623-0.997).

Table 4. The association of diet pattern with abnormal birth outcomes using non-spatial logistical regression*

	Equilibrium pattern			Snacks pattern			Prudent pattern		
	T1	T2	T3	T1	T2	T3	T1	T2	T3
LBW									
Crude OR†	1.032	1	0.797	0.786	1	1.154	0.726	1	0.793
	(0.780-1.366)		(0.587-1.081)	(0.569-1.085)		(0.878-1.516)	(0.544-0.969)		(0.595-1.058)
Adjusted OR††	1.075	1	0.844	0.783	1	1.226	0.732	1	0.788
	(0.800-1.444)		(0.612-1.164)	(0.557-1.100)		(0.919-1.635)	(0.537-0.997)		(0.581-1.069)
Macrosomia									
Crude OR†	1.108	1	1.205	1.207	1	1.237	0.874	1	0.846
	(0.875-1.403)		(0.959-1.515)	(0.942-1.546)		(0.983-1.557)	(0.695-1.098)		(0.673-1.063)
Adjusted OR††	1.137	1	1.169	1.183	1	1.265	0.853	1	0.788

	(0.894-1.446)		(0.925-1.479)	(0.920-1.523)		(1.000-1.602)	(0.674-1.080)		(0.623-0.997)
SGA									
Crude OR†	0.969	1	0.769	0.962	1	1.236	1.006	1	0.956
	(0.816-1.152)		(0.642-0.920)	(0.793-1.166)		(1.042-1.465)	(0.844-1.199)		(0.799-1.143)
Adjusted OR††	0.968	1	0.801	0.996	1	1.26	0.997	1	0.996
	(0.811-1.157)		(0.665-0.965)	(0.817-1.215)		(1.056-1.505)	(0.829-1.198)		(0.828-1.199)
LGA									
Crude OR†	0.946	1	1.087	1.172	1	1.097	0.856	1	0.92
	(0.783-1.142)		(0.907-1.302)	(0.964-1.425)		(0.911-1.321)	(0.711-1.030)		(0.766-1.103)
Adjusted OR††	0.948	1	1.069	1.124	1	1.102	0.836	1	0.88
	(0.782-1.149)		(0.888-1.287)	(0.921-1.373)		(0.911-1.334)	(0.690-1.013)		(0.730-1.062)

T, tertiles; LBW, low birth weight; SGA, small for gestational age; LGA, large for gestational age.

* Values are OR of abnormal birth outcomes and its 95% confidence interval is included in the bracket.

† OR are not adjusted for any variables.

†† OR are adjusted for socio-demographic characteristics (child gender, fetal number, infant parity, childbearing age, mother’s education, mother's residence during pregnancy, Household wealth Index, altitude of residence and area).

Comment [A13]: Table 4. There is no socio-demographic characteristic was shown in this table, is only the dietary pattern that was a significant association with the birth outcome ? kindly add in the complete information in the previous table for socio demographics characteristic for multivariate test

Association between dietary patterns and abnormal birth weight using spatial logistic regression

Based on geographically weighted regression (GWR) for LBW, macrosomia, SGA and LGA in the present study, the optimal bandwidth size were 7332.000, 6631.000, 7596.000, and 7573.000 (persons) respectively. Comparing the global regression and GWR, we concluded that the local regression has better fitness. Approximately 81.6% of mothers with higher scores on the equilibrium pattern was more unlikely to have a SGA infants ($P < 0.05$), with the highest OR observed in North Shaanxi and the lowest OR observed in South Shaanxi. (Table 5 and S5 Fig). The higher scores of snacks pattern during pregnancy was found to be positively associated with LBW for 23.2% of participants ($P< 0.05$), with a downward trend from central Shaanxi to the north and south Shaanxi (Table 5 and S6 Fig). Similarly, infants born to women in the T3 group of snacks

pattern was significantly correlated with macrosomia for 19.5% of participants (P< 0.05), with decreasing OR values from south to north (Table 5 and S7 Fig). Among about 80.0% of participants, all of whom lived in South and Central Shaanxi, there was a higher risk for SGA in the T3 group of snacks pattern than that in the T2 group (P < 0.05). The OR values tend to descend from South to North Shaanxi (Table 5 and S8 Fig). With T2 group of prudent pattern as the reference, a lower risk for LBW among the infants were observed among approximately 75.0% of participants in T1 group and 59.3% of those in T3 group, respectively (P< 0.05). For the OR values of LBW, there was a tendency to decrease from South to North Shaanxi (Table 5, S9 and S10 Figs). The higher scores of prudent pattern was also negatively associated with the increasing risk of fetal macrosomia among 19.2% of participants (P<0.05), with adjusted OR values ranging from 0.875 (South Shaanxi) to 0.939 (Central Shaanxi) (Table 5 and S11 Fig).

Table 5. The association of dietary pattern with abnormal birth outcomes using the GWR model*

	Equilibrium pattern			Snacks pattern			Prudent pattern		
	**T1**	**T2**	**T3**	**T1**	**T2**	**T3**	**T1**	**T2**	**T3**
LBW									
Crude†	0.993(0.895-1.125)	1	0.889(0.742-0.998)	0.886(0.778-0.946)	1	1.126(1.037-1.249)	0.825(0.797-0.919)	1	0.807(0.776-0.967)
Adjusted model††	1.037(0.975-1.135)	1	0.942(0.796-1.007)	0.876(0.819-0.953)	1	1.145(1.066-1.247)	0.814(0.766-0.892)	1	0.835(0.779-0.951)
	(0)		(0)	(6.2)		(23.2)	(75.0)		(59.3)
Macrosomia									
Crude†	1.040(1.029-1.065)	1	1.104(1.073-1.143)	1.080(1.019-1.132)	1	1.099(1.009-1.162)	0.934(0.904-0.950)	1	0.940(0.921-0.965)
Adjusted model††	1.045(0.985-1.089)	1	1.099(1.037-1.149)	1.017(0.969-1.161)	1	1.076(1.027-1.212)	0.929(0.889-0.957)	1	0.912(0.875-0.939)
	(0)		(0)	(6.6)		(19.5)	(0)		(19.2)
SGA									

Crude†	0.997(0.827-1.103)	1	0.836(0.740-1.073)	0.983(0.878-1.099)	1	1.150(0.936-1.235)	1.045(0.962-1.088)	1	0.960(0.875-1.192)
Adjusted model††	0.989(0.972-1.053)	1	0.890(0.883-0.985)	1.007(0.996-1.015)	1	1.104(1.048-1.112)	0.978(0.970-0.985)	1	0.995(0.984-1.004)
	(0)		(81.6)	(0)		(80.0)	(0)		(0)
LGA									
Crude†	0.988(0.927-0.998)	1	1.064(1.035-1.102)	1.092(0.981-1.120)	1	1.052(0.973-1.078)	0.924(0.908-0.962)	1	0.958(0.954-0.987)
Adjusted model††	0.985(0.932-0.999)	1	1.053(1.015-1.097)	1.070(0.962-1.097)	1	1.057(0.988-1.079)	0.927(0.914-0.933)	1	0.934(0.931-0.959)
	(0)		(0)	(0)		(0)	(0)		(0)

T, tertiles; LBW, low birth weight; SGA, small for gestational age; LGA, large for gestational age.

* Values are OR of abnormal birth outcomes and its maximum and minimum are included in the bracket.

† OR are not adjusted for any variables.

†† OR are adjusted for socio-demographic characteristics (child gender, fetal number, infant parity, childbearing age, mother’s education, mother's residence during pregnancy, Household wealth Index and altitude of residence).

Comment [A14]: How is it far different using nonspatial logistic regression and GWR?

Discussion

In the present study, three dietary patterns (equilibrium pattern, prudent pattern and snacks pattern) were identified among the mothers during pregnancy in Shaanxi province of northwestern China. We use spatial regression models to explore the geographic variation of maternal dietary patterns in relation to the risk of abnormal birth weight after controlling for other baseline characteristics.

Previous studies suggested that the single nutrient analysis in nutritional epidemiology failed to account for interactions between food components or foods. Dietary patterns represent a broader picture of food and nutrient intakes and are able to account for cumulative and interactive effects [24]. The results suggested that

pregnant women with high adherence to the equilibrium pattern tended to eat a diet with a balance of all of the food groups and foods which is rich in energy and various nutrients. Moreover, participants with high adherence to the equilibrium pattern were more likely to be better educated, wealthier, 25–29 years old at delivery, and living at lower altitudes. The characteristics of this dietary model identified in our study was homologous to the dietary patterns in Guangzhou, China [25]. The prudent pattern was characterized by high intakes of dairy products and eggs, and the snacks pattern had high loading from beverages, sweetmeat, and fast-food. Those with higher scores on the prudent pattern and the snacks pattern had lower energy intake, carbohydrate intake and lower intakes of some selected nutrients. The sociodemographic distribution of the prudent pattern was similar to the equilibrium pattern, while those with high scores on the snacks pattern were more likely to live in higher altitudes areas (>1000 meters), be poor and less educated, and be aged 18-24 years at delivery. Some studies had reported that higher education level and better economic status might enable individuals to make healthy diet choices [26,27]. Hence, people with higher education level and better economic status tend to be more inclined to the equilibrium pattern [28,29,30,31]. Conversely, low socioeconomic groups with relatively low level of nutrition knowledge may increase ability for making unhealthy diet choices and instead lead to choosing a poor-quality snacks pattern [32].

Due to the huge difference in geographical environment and socio-culture across various areas in China, it was well-recognized that the dietary habits and food consumption have different geographical distribution [33,34]. Similarly, dietary habits varied largely among different areas in Shaanxi [35]. In our studies, the scores of the equilibrium pattern were highest in Central Shaanxi and lowest in North Shaanxi. The scores of the snacks pattern were higher in North Shaanxi, followed by South Shaanxi and Central Shaanxi. It appeared a decreasing trend of the scores of the prudent pattern from south to north. It is possible that the different geographical environment (e.g. the area altitudes) resulted in different geographical distribution of dietary

patterns.

Comment [A15]: Line 307. Does it mean the different patterns mostly influenced by altitude? Why didn't the author show others based on the rural and urban type of area?

Consuming a healthy diet characterized by a variety of nutritious foods during pregnancy is essential for promoting and maintaining fetal health [29,36]. Global regression analysis revealed that mothers with high adherence to the equilibrium pattern during pregnancy were more unlikely to give birth to a baby with SGA. The findings suggested that equilibrium pattern with a balance of all of the food groups and foods, may help to reduce the risk for SGA, which provided more evidence of the benefits of healthy diets on birth weight. Further, the GWLR model showed that equilibrium pattern might reduce more neonates with SGA in Central and South Shaanxi than that in North Shaanxi. Our survey data indicated that the scores of equilibrium pattern was higher in Central and South Shaanxi than in North Shaanxi. Most parts of Central Shaanxi are developed urban areas (e.g., Xi 'an), where mothers have higher household economic levels and higher education. In contrast, the surveyed area in North Shaanxi comprise small townships and villages, where mothers have lower economic levels and less educated. The greater the density of villages, the more likely the villagers are to eat high-starch foods, and the more frugal the dietary structure tends to be [37]. These results remind us that it is extremely urgent to advocate the equilibrium diet during pregnancy associated with appropriate birth weight, especially in North Shaanxi. Additional studies will be required to corroborate these findings and to elucidate possible reasons for this geographical variations.

There is a high intake of snack food among the Chinese in recent years [38]. The snacks foods as an unhealthy food choice is unable to fulfill the increased key micronutrients requirements for normal fetal growth and may lead to a higher risk of some diseases [39]. According to the global regression, the higher scores of snacks pattern was associated with the increasing risk for macrosomia and SGA among the infants. The snacks pattern consists of foods with high concentrations of simple carbohydrates, lipids and low amounts of protein and micronutrients. Insufficient protein intake during pregnancy will affect fetal organ development,

leading to a variety of adverse pregnancy outcomes [40].During pregnancy, fetal growth and development are very rapid, and inadequate nutrition intake will lead to fetal growth retardation and fetal low birth weight [41].However, some studies also found that this combination of substances in the snacks pattern may lead to rapid fetal weight gain and increase the incidence of macrosomia [42]. Therefore, the snacks pattern gives these foods unbalanced nutrients contents, favoring abnormal birth weight [43,44].

Further, the GWLR model revealed that snacks pattern during pregnancy may increase the risk for LBW, Macrosomia and SGA in 23.2%, 19.5% and 80.0%, respectively of all participants. When we focus on the map drawn by OR values, it is easy to see that OR values of LBW were highest in Central Shaanxi, and OR values of Macrosomia and SGA progressively decreased from South to North Shaanxi. Thus, it is essential to improve the maternal diet status during pregnancy according to regional characteristics.

The spatial regression model revealed a negative association between high propensity of prudent pattern and the risk of LBW for 59.3% of mothers residing in Central Shaanxi and parts of North Shaanxi. On the map used to visualize these results, prudent pattern might decrease more neonates with LBW in North Shaanxi, followed by Central Shaanxi. The negative association of prudent pattern with macrosomia were mainly distributed in South Shaanxi. Due to the prudent pattern with high intakes of dairy products and eggs, which included higher protein and micronutrients compared to snacks pattern, it might benefit maintaining appropriate birth weight. However, the geographical variations after controlling for the important risk factors provide potential policy implications, calling for further investigations.

However, we have to acknowledge some limitations needed to address in the study. First, due to the cross-sectional design of our study, we cannot draw any cause-effect conclusion based on the study results. Secondly, it was difficult to isolate the variation trends in relevant factors based on limited number of areas sampled in our study. Thirdly, the mothers' self-reported information on dietary intake during pregnancy was adopted.

Thus, the possibility of information bias should be considered. Fourthly, the study may be subject to some potential confounders. For example, drug use, genetics, and environmental factors were not available and thus could not be controlled in multivariate analysis. Despite these limitations, this study has still provided important information on the geographic varying association of dietary patterns with abnormal birth weight in northwestern China, and filled in some of the gaps.

Comment [A16]: Line 354. Birth weight outcomes have close relation with the BMI of the mother (nutrition status of pregnant). Did the author consider this variable in their analysis or take in the limitation study.

Conclusions

In summary, three dietary patterns (equilibrium pattern, prudent pattern and snacks pattern) were extracted among the mothers during pregnancy, which had different geographical distribution in Shaanxi province of northwestern China. The equilibrium pattern, which is rich in energy and various nutrients, was negatively associated with SGA. The prudent pattern were more likely to decrease the risk of LBW and macrosomia, respectively mainly in North Shaanxi and South Shaanxi. The snacks pattern during pregnancy may cause abnormal birth weight (e.g. LBW, macrosomia, SGA) among infants, especially in Central and South Shaanxi. The findings emphasize the importance of geographical distribution to improve the dietary patterns among disadvantaged pregnant women.

Acknowledgements

We thank the women and their children participated in our study. We thank local hospitals and health bureau as well as the Ministry of Health in Shaanxi province.

References

1. Stephens TV, Woo H, Innis SM, Elango R. Healthy pregnant women in Canada are consuming more dietary protein at 16- and 36-week gestation than currently recommended by the Dietary Reference Intakes, primarily from dairy food sources. Nutr Res. 2014 Jul;34(7):569-576. doi: 10.1016/j.nutres.2014.07.001. Epub 2014 Jul

8. PMID: 25150115.

2. Cetin I, Berti C, Calabrese S. Role of micronutrients in the periconceptional period. Hum Reprod Update. 2010 Jan-Feb;16(1):80-95. doi: 10.1093/humupd/dmp025. PMID: 19567449.

3. Wrottesley SV, Lamper C, Pisa PT. Review of the importance of nutrition during the first 1000 days: maternal nutritional status and its associations with fetal growth and birth, neonatal and infant outcomes among African women. J Dev Orig Health Dis. 2016 Apr;7(2):144-162. doi: 10.1017/S2040174415001439. Epub 2015 Aug 17. PMID: 26279311.

4. Paknahad Z, Fallah A, Moravejolahkami AR. Maternal Dietary Patterns and Their Association with Pregnancy Outcomes. Clin Nutr Res. 2019 Jan 25;8(1):64-73. doi: 10.7762/cnr.2019.8.1.64. PMID: 30746349; PMCID: PMC6355947.

5. Hu FB. Dietary pattern analysis: a new direction in nutritional epidemiology. Curr Opin Lipidol. 2002 Feb;13(1):3-9. doi: 10.1097/00041433-200202000-00002. PMID: 11790957.

6. Völgyi E, Carroll KN, Hare ME, Ringwald-Smith K, Piyathilake C, Yoo W, et al. Dietary patterns in pregnancy and effects on nutrient intake in the Mid-South: the Conditions Affecting Neurocognitive Development and Learning in Early Childhood (CANDLE) study. Nutrients. 2013 May 3;5(5):1511-1530. doi: 10.3390/nu5051511. PMID: 23645026; PMCID: PMC3708333.

7. Thompson JM, Wall C, Becroft DM, Robinson E, Wild CJ, Mitchell EA. Maternal dietary patterns in pregnancy and the association with small-for-gestational-age infants. Br J Nutr. 2010 Jun;103(11):1665-1673. doi: 10.1017/S0007114509993606. Epub 2010 Mar 9. PMID: 20211035.

8. Northstone K, Emmett PM, Rogers I. Dietary patterns in pregnancy and associations with nutrient intakes. Br J Nutr. 2008 Feb;99(2):406-415. doi: 10.1017/S0007114507803977. Epub 2007 Sep 3. PMID: 17764600; PMCID: PMC2492390.

9. Bukowski R, Hansen NI, Willinger M, Reddy UM, Parker CB, Pinar H, et al. Eunice Kennedy Shriver National Institute of Child Health and Human Development Stillbirth Collaborative Research Network. Fetal growth and risk of stillbirth: a population-based case-control study. PLoS Med. 2014 Apr 22;11(4):e1001633. doi: 10.1371/journal.pmed.1001633. PMID: 24755550; PMCID: PMC3995658.

10. Grissom NM, Reyes TM. Gestational overgrowth and undergrowth affect neurodevelopment: similarities and differences from behavior to epigenetics. Int J Dev Neurosci. 2013 Oct;31(6):406-414. doi: 10.1016/j.ijdevneu.2012.11.006. Epub 2012 Nov 28. PMID: 23201144; PMCID: PMC6288817.

11. Wennerström EC, Simonsen J, Melbye M. Long-Term Survival of Individuals Born Small and Large for Gestational Age. PLoS One. 2015 Sep 21;10(9):e0138594. doi: 10.1371/journal.pone.0138594. PMID: 26390219; PMCID: PMC4577072.

12. Francis JH, Permezel M, Davey MA. Perinatal mortality by birthweight centile. Aust N Z J Obstet Gynaecol. 2014 Aug;54(4):354-359. doi: 10.1111/ajo.12205. Epub 2014 Apr 15. PMID: 24731210.

13. Pei L, Kang Y, Zhao Y, Yan H. Prevalence and risk factors of congenital heart defects among live births: a population-based cross-sectional survey in Shaanxi province, Northwestern China. BMC Pediatr. 2017 Jan 13;17(1):18. doi: 10.1186/s12887-017-0784-1. PMID: 28086762; PMCID: PMC5237335.

14. Cheng Y, Dibley MJ, Zhang X, Zeng L, Yan H. Assessment of dietary intake among pregnant women in a rural area of western China. BMC Public Health. 2009 Jul 9;9:222. doi: 10.1186/1471-2458-9-222. PMID: 19589154; PMCID: PMC2716336.

15. Cheng Y, Yan H, Dibley MJ, Shen Y, Li Q, Zeng L. Validity and reproducibility of a semi-quantitative food frequency questionnaire for use among pregnant women in rural China. Asia Pac J Clin Nutr. 2008;17(1):166-177. PMID: 18364342.

16. Institute of Nutrition and Food Safety, China Center for Disease Control and Prevention (2009) China

Food Composition Book 1, 2nd ed. Beijing: Peking University Medical Press.

17. Jie L. Obstetrics and Gynecology [M]. 8 version. Beijing: People's Medical Publishing House; 2013. pp.47–58.

18. Ikem E, Halldorsson TI, Birgisdóttir BE, Rasmussen MA, Olsen SF, Maslova E. Dietary patterns and the risk of pregnancy-associated hypertension in the Danish National Birth Cohort: a prospective longitudinal study. BJOG. 2019 Apr;126(5):663-673. doi: 10.1111/1471-0528.15593. Epub 2019 Jan 24. PMID: 30675768.

19. Schulze MB, Hoffmann K, Kroke A, Boeing H. An approach to construct simplified measures of dietary patterns from exploratory factor analysis. Br J Nutr. 2003 Mar;89(3):409-419. doi: 10.1079/BJN2002778. PMID: 12628035.

20. Shi Z, Hu X, Yuan B, Pan X, Dai Y, Holmboe-Ottesen G. Association between dietary patterns and anaemia in adults from Jiangsu Province in Eastern China. Br J Nutr. 2006 Nov;96(5):906-912. doi: 10.1017/bjn20061785. PMID: 17092381.

21. Yang TC, Matthews SA. Understanding the non-stationary associations between distrust of the health care system, health conditions, and self-rated health in the elderly: a geographically weighted regression approach. Health Place. 2012 May;18(3):576-585. doi: 10.1016/j.healthplace.2012.01.007. Epub 2012 Jan 28. PMID: 22321903; PMCID: PMC3319514.

22. Zhou YB, Wang QX, Liang S, Gong YH, Yang MX, Chen Y, et al. Geographical variations in risk factors associated with HIV infection among drug users in a prefecture in Southwest China. Infect Dis Poverty. 2015 Sep 2;4:38. doi: 10.1186/s40249-015-0073-x. PMID: 26329887; PMCID: PMC4557839.

23. Nakaya T, Fotheringham AS, Brunsdon C, Charlton M. Geographically weighted Poisson regression for disease association mapping. Stat Med. 2005 Sep 15;24(17):2695-2717. doi: 10.1002/sim.2129. PMID: 16118814.

24. Hu FB. Dietary pattern analysis: a new direction in nutritional epidemiology. Curr Opin Lipidol. 2002 Feb;13(1):3-9. doi: 10.1097/00041433-200202000-00002. PMID: 11790957.

25. He JR, Yuan MY, Chen NN, Lu JH, Hu CY, Mai WB, et al. Maternal dietary patterns and gestational diabetes mellitus: a large prospective cohort study in China. Br J Nutr. 2015 Apr 28;113(8):1292-1300. doi: 10.1017/S0007114515000707. Epub 2015 Mar 30. PMID: 25821944.

26. Mi W, Lian W, Yang M, Jiang Y, Yi W, Wang Y, et al. Analysis on dietary patterns and influencing factors among the elderly in Yantai City. Wei Sheng Yan Jiu. 2017 Jan;46(1):27-31. Chinese. PMID: 29903147.

27. Luksiene DI, Baceviciene M, Tamosiunas A, Daugeliene E, Kranciukaite D. Health, alcohol and psychosocial factors in Eastern Europe study: dietary patterns and their association with socio-demographic factors in the Lithuanian urban population of Kaunas city. Int J Public Health. 2011 Apr;56(2):209-216. doi: 10.1007/s00038-010-0170-3. Epub 2010 Aug 12. PMID: 20703510; PMCID: PMC3947489.

28. Carniel EF, Zanolli ML, Antonio MARGM, Morcillo AM. Determinantes do baixo peso ao nascer a partir das Declarações de Nascidos Vivos.[J]. Revista Brasileira de Epidemiologia. 2008;11(1):169–179. doi:10.1590/S1415-790X2008000100016.

29. Cucó G, Fernández-Ballart J, Sala J, Viladrich C, Iranzo R, Vila J, et al. Dietary patterns and associated lifestyles in preconception, pregnancy and postpartum. Eur J Clin Nutr. 2006 Mar;60(3):364-71. doi: 10.1038/sj.ejcn.1602324. PMID: 16340954.

30. Ferrer C, Garcia-Esteban R, Mendez M, Robieu I, Sunyer MTJ. Determinantes sociales de los patrones dietéticos durante el embarazo[J]. Gaceta Sanitaria. 2009;23(1):38–43. doi: 10.1016/j.gaceta.2008.05.004

31. Northstone K, Emmett P, Rogers I. Dietary patterns in pregnancy and associations with socio-demographic and lifestyle factors. Eur J Clin Nutr. 2008 Apr;62(4):471-479. doi: 10.1038/sj.ejcn.1602741. Epub 2007 Mar 21. PMID: 17375108; PMCID: PMC2492391.

32. Taylor MK, Sullivan DK, Ellerbeck EF, Gajewski BJ, Gibbs HD. Nutrition literacy predicts adherence to healthy/unhealthy diet patterns in adults with a nutrition-related chronic condition. Public Health Nutr. 2019 Aug;22(12):2157-2169. doi: 10.1017/S1368980019001289. Epub 2019 May 31. PMID: 31146797; PMCID: PMC6827561.

33. Cheng, C.K. The culture of Chinese diet: Regional differentiation and developing trends[J]. Acta Geographica Sinica, 1994, 49(3): 226-235.

34. Song F, Cho MS. Geography of Food Consumption Patterns between South and North China. Foods. 2017 May 5;6(5):34. doi: 10.3390/foods6050034. PMID: 28475146; PMCID: PMC5447910.

35. Wang J, Lin X, Bloomgarden ZT, Ning G. The Jiangnan diet, a healthy diet pattern for Chinese. J Diabetes. 2020 May;12(5):365-371. doi: 10.1111/1753-0407.13015. Epub 2020 Jan 26. PMID: 31846221; PMCID: PMC7216939.

36. Thompson JM, Wall C, Becroft DM, Robinson E, Wild CJ, Mitchell EA. Maternal dietary patterns in pregnancy and the association with small-for-gestational-age infants. Br J Nutr. 2010 Jun;103(11):1665-1673. doi: 10.1017/S0007114509993606. Epub 2010 Mar 9. PMID: 20211035.

37. Hajianfar H, Esmaillzadeh A, Feizi A, Shahshahan Z, Azadbakht L. The Association Between Major Dietary Patterns and Pregnancy-related Complications. Arch Iran Med. 2018 Oct 1;21(10):443-451. PMID: 30415552.

38. Wang D, He Y, Li Y, Luan D, Yang X, Zhai F, et al. Dietary patterns and hypertension among Chinese adults: a nationally representative cross-sectional study. BMC Public Health. 2011 Dec 14;11:925. doi: 10.1186/1471-2458-11-925. PMID: 22168909; PMCID: PMC3299712.

39. Shang X, Li Y, Liu A, Zhang Q, Hu X, Du S, et al. Dietary pattern and its association with the prevalence of obesity and related cardiometabolic risk factors among Chinese children. PLoS One. 2012;7(8):e43183.

doi: 10.1371/journal.pone.0043183. Epub 2012 Aug 14. PMID: 22905228; PMCID: PMC3419173.

40. Flynn AC, Schneeberger C, Seed PT, Barr S, Poston L, Goff LM. The Effects of the UK Pregnancies Better Eating and Activity Trial Intervention on Dietary Patterns in Obese Pregnant Women Participating in a Pilot Randomized Controlled Trial. Nutr Metab Insights. 2016 Jun 27;8(Suppl 1):79-86. doi: 10.4137/NMI.S29529. PMID: 27385914; PMCID: PMC4924882.

41. Diemert A, Lezius S, Pagenkemper M, Hansen G, Drozdowska A, Hecher K, et al. Maternal nutrition, inadequate gestational weight gain and birth weight: results from a prospective birth cohort. BMC Pregnancy Childbirth. 2016 Aug 15;16:224. doi: 10.1186/s12884-016-1012-y. PMID: 27528213; PMCID: PMC4986204..

42. Flynn AC, Seed PT, Patel N, Barr S, Bell R, Briley AL, et al. UPBEAT consortium. Dietary patterns in obese pregnant women; influence of a behavioral intervention of diet and physical activity in the UPBEAT randomized controlled trial. Int J Behav Nutr Phys Act. 2016 Nov 29;13(1):124. doi: 10.1186/s12966-016-0450-2. PMID: 27894316; PMCID: PMC5126873.

43. Coelho Nde L, Cunha DB, Esteves AP, Lacerda EM, Theme Filha MM. Dietary patterns in pregnancy and birth weight. Rev Saude Publica. 2015;49:62. doi: 10.1590/S0034-8910.2015049005403. Epub 2015 Sep 18. PMID: 26398873; PMCID: PMC4617437.

44. Uusitalo U, Arkkola T, Ovaskainen ML, Kronberg-Kippilä C, Kenward MG, Veijola R, et al. Unhealthy dietary patterns are associated with weight gain during pregnancy among Finnish women. Public Health Nutr. 2009 Dec;12(12):2392-2399. doi: 10.1017/S136898000900528X. Epub 2009 Mar 27. PMID: 19323867.

Supporting information

S1 Fig. Geographical distribution of the incidence of LBW among the live infants in Shaanxi province, 2013.

S2 Fig. Geographical distribution of the incidence of macrosomia among the live infants in Shaanxi province, 2013.

S3 Fig. Geographical distribution of the incidence of SGA among the live infants in Shaanxi province, 2013.

S4 Fig. Geographical distribution of the incidence of LGA among the live infants in Shaanxi province, 2013.

S5 Fig. Geographical distribution of adjusted ORs of equilibrium pattern of T3, as associated with prevalence of SGA.

S6 Fig. Geographical distribution of adjusted ORs of snacks pattern of T3, as associated with prevalence of LBW.

S7 Fig. Geographical distribution of adjusted ORs of snacks pattern of T3, as associated with prevalence of macrosomia.

S8 Fig. Geographical distribution of adjusted ORs of snacks pattern of T3, as associated with prevalence of SGA.

S9 Fig. Geographical distribution of adjusted ORs of prudent pattern of T1, as associated with prevalence of LBW.

S10 Fig. Geographical distribution of adjusted ORs of prudent pattern of T3, as associated with prevalence of LBW.

S11 Fig. Geographical distribution of adjusted ORs of prudent pattern of T3, as associated with prevalence of macrosomia.

S1 File. Informed consent.

Figure

Click here to access/download;Figure;Fig 1.tif

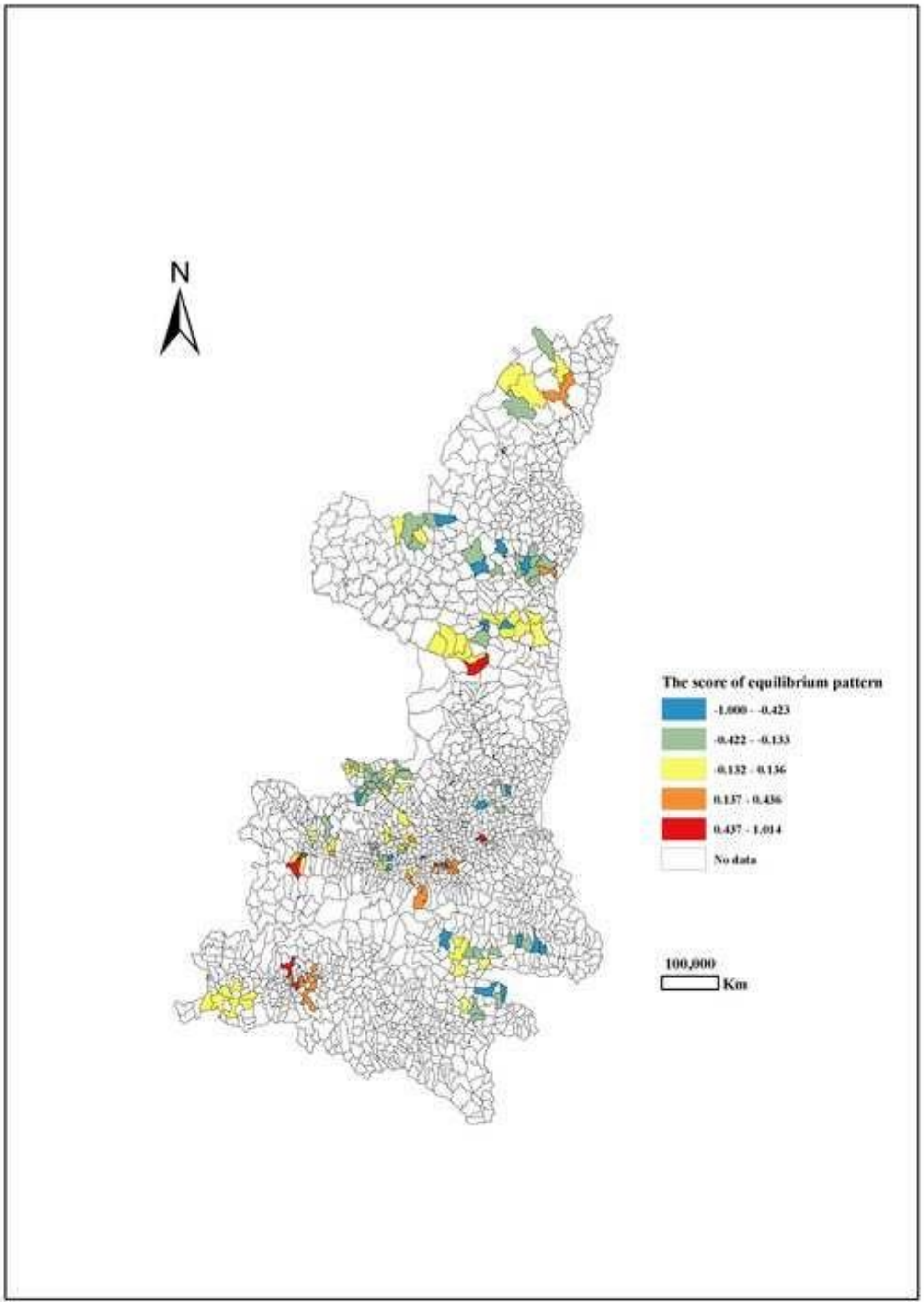

Click here to access/download;Figure;Fig 2.tif

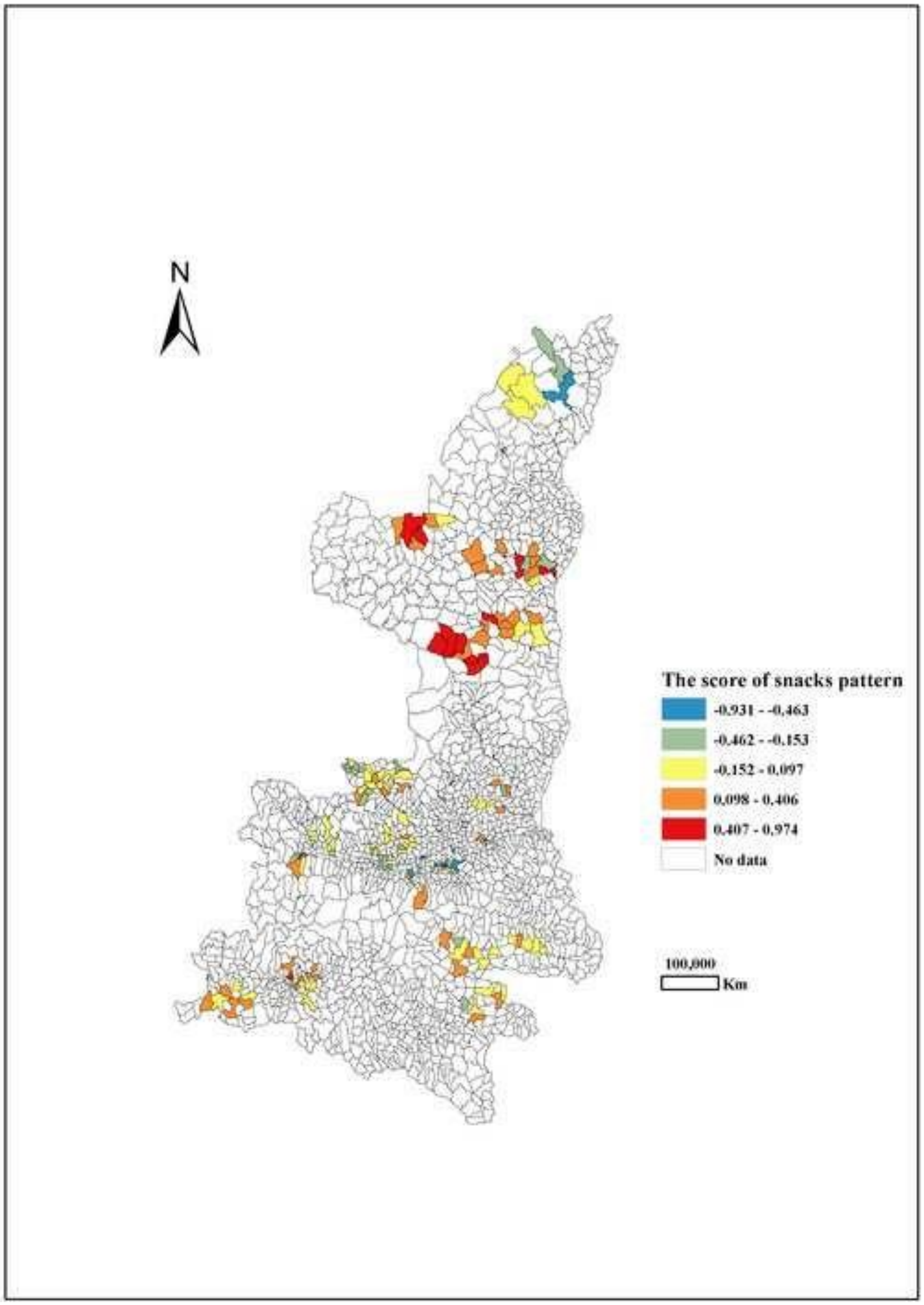

Click here to access/download;Figure;Fig 3.tif

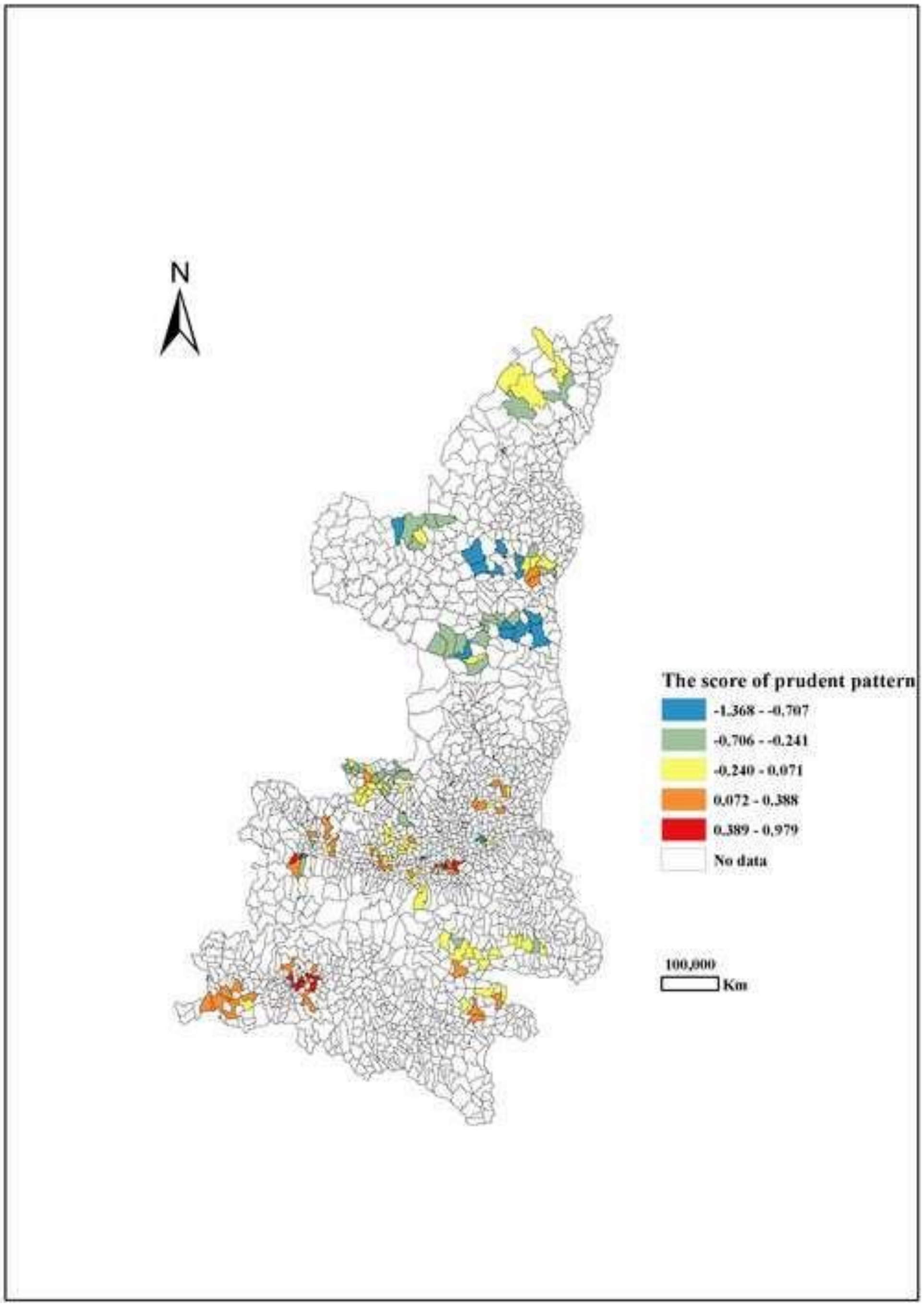

Supporting Information

Click here to access/download
Supporting Information
Informed consent.doc

Click here to access/download
Supporting Information
S1 Fig.tif

Click here to access/download
Supporting Information
S2 Fig.tif

Click here to access/download
Supporting Information
S3 Fig.tif

Click here to access/download
Supporting Information
S4 Fig.tif

Click here to access/download
Supporting Information
S5 Fig.tif

Click here to access/download
Supporting Information
S6 Fig.tif

Click here to access/download
Supporting Information
S7 Fig.tif

Click here to access/download
Supporting Information
S8 Fig.tif

Click here to access/download
Supporting Information
S9 Fig.tif

Click here to access/download
Supporting Information
S10 Fig.tif

Click here to access/download
Supporting Information
S11 Fig.tif

Database

Click here to access/download
Supporting Information
Dietary pattern Database.sav
